# Integrative Physiological and Transcriptomic Analyses of Oat (*Avena sativa* L.) Seedlings Under Severe Salt Stress

**DOI:** 10.3390/plants15172712

**Published:** 2026-09-03

**Authors:** Rui Qiu, Xinyi Zhang, Xiangpeng Kong, Wenjie Zhao, Qianhan Zhao, Yingying Chu, Muzhapaer Tuluohong, Baiji Wang, Guowen Cui, Bing Li

**Affiliations:** Department of Grassland Science, College of Animal Science and Technology, Northeast Agricultural University, Harbin 150030, China; qr020327@163.com (R.Q.); zzxxyy020509@163.com (X.Z.); kl18242446011@163.com (X.K.); 13310467886@163.com (W.Z.); b2505029016@neau.edu.cn (Q.Z.); m15336343174@163.com (Y.C.); 17645096163@163.com (M.T.); wbj011211@163.com (B.W.)

**Keywords:** oat, salt stress, physiological responses, comparative transcriptomics, WGCNA

## Abstract

Crop productivity around the world is largely constrained by salt-induced stress, a key abiotic factor. Although oat (*Avena sativa* L.) can withstand challenging environmental conditions, the physiological and molecular responses underlying salt tolerance during germination and early seedling development remain insufficiently understood. To investigate these responses, 28 oat varieties were evaluated at the germination stage, and two contrasting varieties, the salt-tolerant Mengshi No. 1 (MS) and salt-sensitive Morgan (MG), were selected for detailed analysis under a severe NaCl treatment (300 mM) during early seedling stages. Under severe salt stress, the two oat varieties exhibited distinct growth and physiological responses, including changes in growth traits, chlorophyll content, membrane stability, osmotic adjustment, and antioxidant responses. Transcriptomic analysis revealed 14,109 differentially expressed genes (DEGs) between salt-treated MG and its respective control (CK), 19,405 between salt-treated MS and its CK, and 6161 between salt-treated MG and salt-treated MS, suggesting different transcriptional response patterns between the salt-tolerant and salt-sensitive varieties under severe salt stress. Weighted gene co-expression network analysis (WGCNA) revealed a salt-responsive module associated with MS, from which five hub genes, *AVESA.00010b.r2.1CG0087930* (*MGL*), *AVESA.00010b.r2.19DG0180280* (*MGL*), *AVESA.00010b.r2.4CG1272260* (*BCH1*), *AVESA.00010b.r2.5DG0989800* (*GPAT7*), and *AVESA.00010b.r2.6CG1124100* (*TPR10*), were identified as candidate genes potentially associated with salt tolerance and stress responses.

## 1. Introduction

Soil salinization constitutes a critical global challenge, substantially reducing crop productivity and constraining land utilization [1]. In China, approximately 76 million hectares are affected by saline–alkali soils [2]. Salt stress facilitates the accumulation of toxic ions, induces reactive oxygen species formation, impairs photosynthetic performance, compromises membrane integrity, and perturbs osmotic regulation within plant cells [3]. To mitigate these adverse impacts, plants have evolved sophisticated salt-tolerance strategies, including the accumulation of osmotic regulators, the preservation of ion homeostasis, the activation of antioxidant enzymes, and the precise modulation of gene expression [4].

Oats (*Avena sativa* L.), belonging to the Poaceae family, are annual herbs exhibiting strong tolerance to salinity, cold, drought, and infertile soils, along with broad ecological adaptability [5]. Oats are nutritionally valuable and display relatively strong salt tolerance among cereal crops [6]. Oat cultivation is concentrated in the Northeast, North, and Northwest regions, where large areas of farmland are affected by salinization and secondary salinization in China [7]. In the black soil region of Northeast China alone, nearly 3.7 million hectares of low- and medium-yield farmland in the central and western Songnen Plain are affected by salinity [8]. Although oats tolerate moderate salinity, excessively high soil salinity can still reduce both yield and quality. Investigating oat salt tolerance supports the development of varieties adapted to saline–alkali environments and facilitates the improvement and utilization of saline–alkali lands [9]. Early investigations into oat salt stress primarily concentrated on screening and evaluating germplasm resources during the germination and early seedling stages [6,10,11]. Concurrently, considerable attention was devoted to examining the responses of diverse physiological and biochemical indicators, such as photosynthetic parameters, to salt stress [12,13,14]. Recognizing the inherent limitations of relying on isolated parameters, recent screening protocols have increasingly integrated multi-criteria comprehensive evaluation methods [15,16]. More recently, the advent of high-throughput technologies has enabled researchers to dissect the molecular architecture of oat salt stress through multi-omics approaches [5,17,18]. However, systematic analyses elucidating the complex regulatory networks underlying oat salt tolerance remain limited, hindering a comprehensive understanding of the mechanisms and their application in breeding programs.

Advanced high-throughput approaches, particularly RNA sequencing (RNA-seq), have greatly facilitated comprehensive functional analyses of genes and are extensively utilized to examine responses to salinity stress in species [19]. Although several transcriptomic investigations have been performed in oat, the molecular mechanisms and regulatory networks underlying salinity adaptation are still insufficiently understood. Weighted gene co-expression network analysis (WGCNA), a systems biology approach widely employed to evaluate gene co-expression patterns, has proven effective for identifying biologically meaningful modules [20]. Transcriptome studies across multiple plants have extensively applied this method to elucidate essential regulatory networks and potential genes conferring salinity tolerance [21,22].

In the present study, salt-tolerant and salt-sensitive oat varieties were identified based on germination parameters of 28 varieties under salt stress. Growth, physiological, and transcriptomic responses were further evaluated in the selected varieties under severe NaCl stress (300 mM) during the early seedling stage. By integrating physiological and transcriptomic data, WGCNA identified hub genes potentially associated with salt stress responses. This study provides insights into the physiological and transcriptional responses of oat seedlings under severe salt stress. The findings enhance our understanding of oat responses to salinity and provide transcriptomic resources for further investigation of candidate genes associated with salt stress responses.

## 2. Results

### 2.1. Screening and Evaluation of Salt Tolerance in Oat Varieties

**Figure 1:**
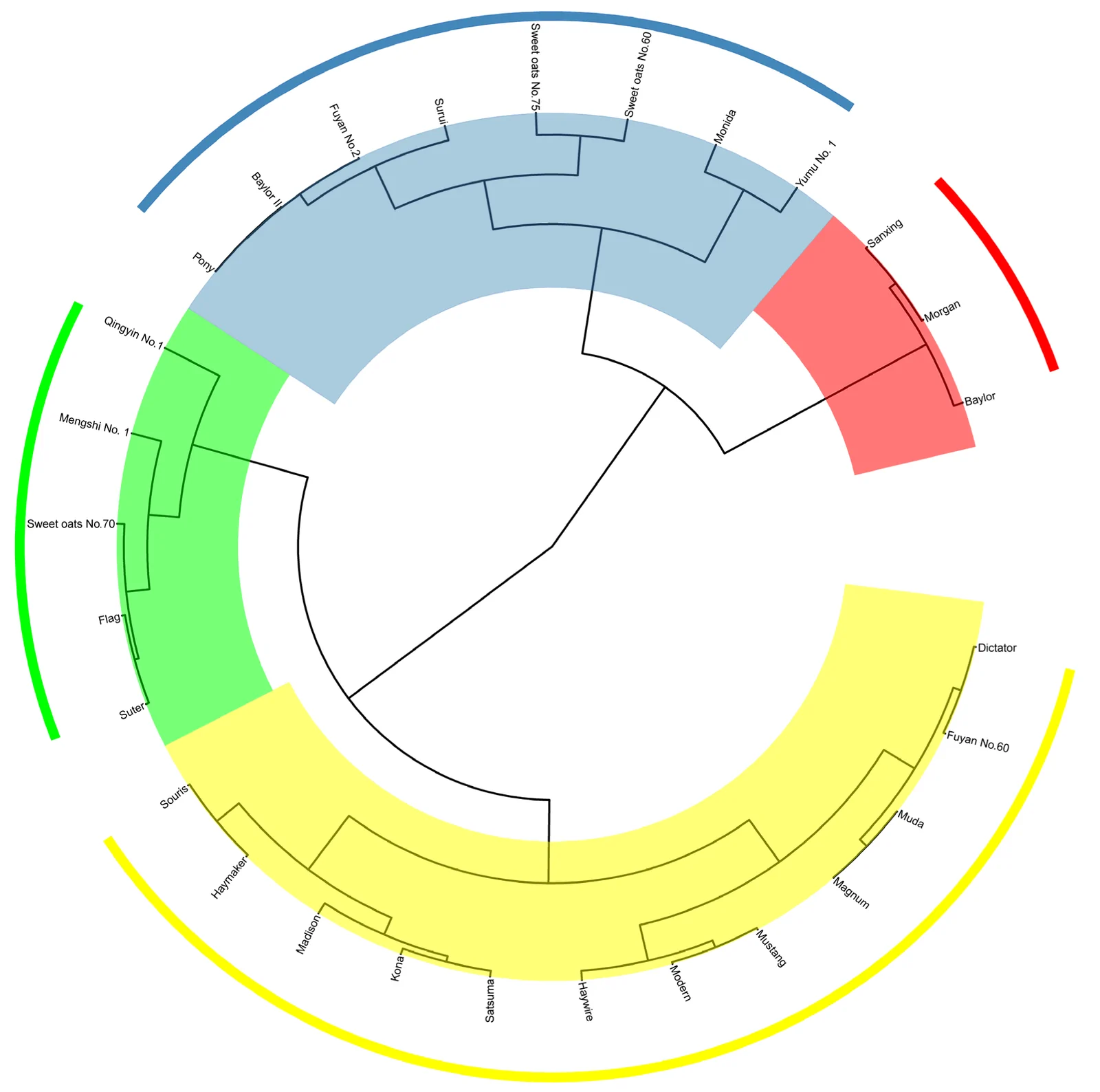
Cluster analysis of the relative values of 28 varieties and 5 measured traits. Different colors represent different salt-tolerance categories.

To assess salt tolerance in 28 oat varieties, root length, shoot length, and germination traits (germination rate, GR; germination energy, GE; germination index, GI) were used as indicators. Principal component analysis (PCA) was performed on the relative values to select two comprehensive indicators with eigenvalues greater than 1, followed by membership function calculation and cluster analysis based on the *D*-values (Table S1–S5). Based on a comprehensive evaluation, the varieties were grouped into four categories, with Mengshi No. 1 (MS) identified as salt-tolerant and Morgan (MG) as salt-sensitive (Figure 1).

### 2.2. Phenotypic and Growth Responses of Oat Varieties to Salt Stress

**Figure 2:**
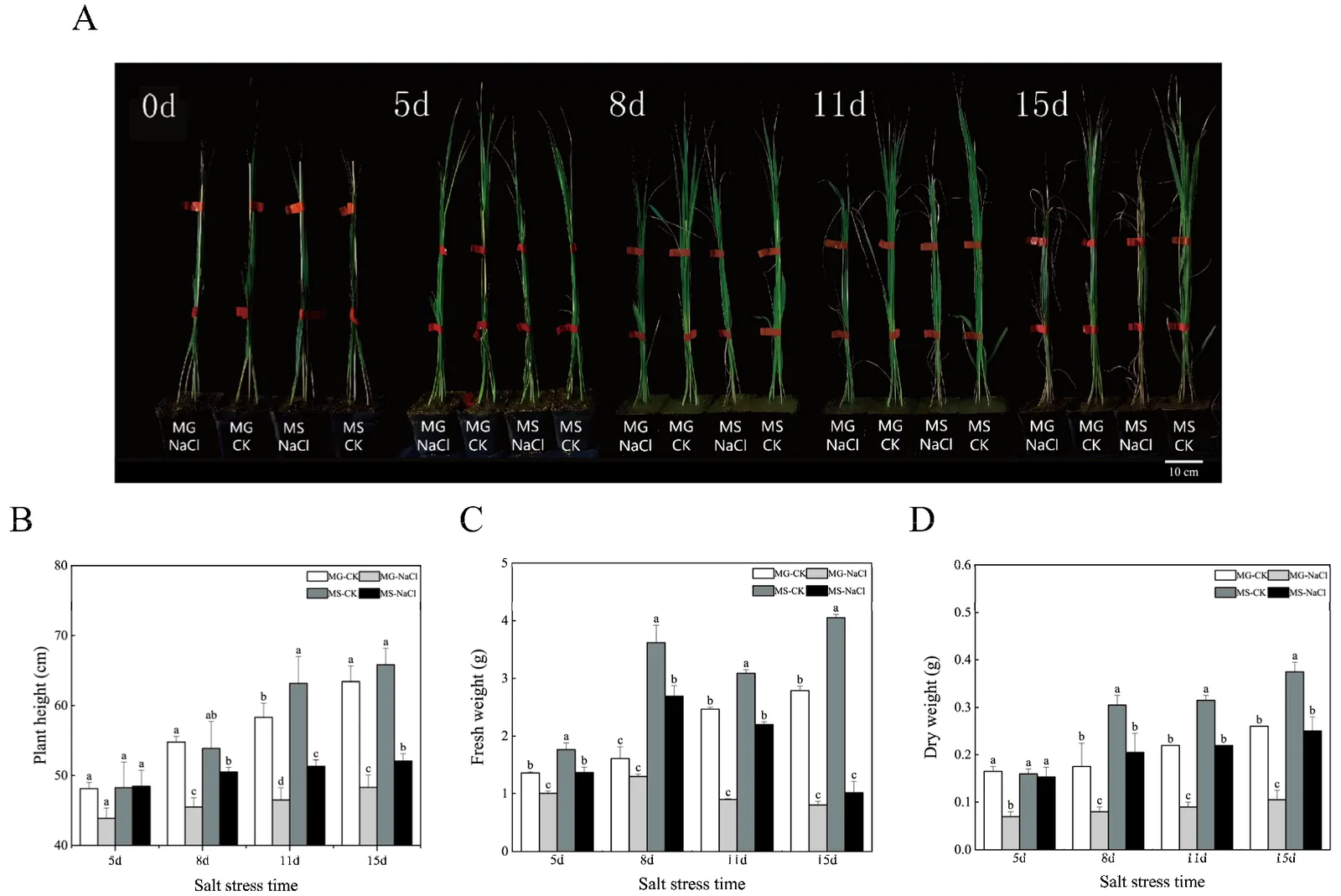
Phenotypic changes (**A**), plant height (**B**), fresh weight (**C**), and dry weight (**D**) of two oat varieties (MS and MG) subjected to salt stress for 5, 8, 11, and 15 d. Values are mean ± SD. Different lowercase letters indicate significant differences among the variety × salt treatment combinations within each sampling time point (*p* < 0.05).

Phenotypic differences between the two oat varieties became increasingly evident throughout the treatment period. After 5 d of treatment, both varieties maintained relatively normal growth characteristics, with no obvious visible differences compared with CK. By day 8, slight leaf wilting and chlorosis were observed, with MG exhibiting more pronounced stress symptoms than MS. At day 11, both treated varieties displayed clear morphological alterations, including leaf drooping, wilting, and chlorosis; however, MS retained a relatively upright growth habit. After 15 d of treatment, both varieties exhibited pronounced wilting and chlorosis, while MG showed greater morphological deterioration than MS (Figure 2A).

The three-way ANOVA revealed highly significant effects of oat variety, salt treatment, and time on plant height, fresh weight, and dry weight (*p* < 0.001). The variety × salt treatment interaction significantly affected plant height and dry weight (*p* < 0.05) and fresh weight (*p* < 0.01). The variety × time interaction exerted highly significant effects on fresh weight and dry weight (*p* < 0.001), while no significant effect was observed for plant height. The salt treatment × time interaction significantly affected all three traits (plant height and fresh weight, *p* < 0.001; dry weight, *p* < 0.01). The three-way interaction significantly affected only fresh weight (*p* < 0.001) (Table 1). Under both normal and salt stress conditions, seedling height of both oat varieties increased with cultivation time. As the duration of salt stress increased, plant height growth was inhibited to different extents in the two varieties (Figure 2B–D). At 11 d and 15 d, plant heights of salt-stressed MS and MG were significantly lower than those of their corresponding control (CK) (*p* < 0.05). At 15 d, the difference in plant height between salt treatment and CK reached its maximum; salt-stressed MG and MS exhibited height reductions of 23.82% and 20.96%, respectively, relative to their corresponding CK groups. After 15 d of salt stress, fresh weight (FW) and dry weight (DW) decreased significantly compared with the corresponding CK groups. In MG, FW and DW decreased by 71.05% and 59.62%, respectively (*p* < 0.05). In MS, FW and DW decreased by 74.90% and 33.33%, respectively (*p* < 0.05).

### 2.3. Physiological Responses of Oat Varieties to Salt Stress

#### 2.3.1. Changes in Antioxidant Enzyme Activity

**Figure 3:**
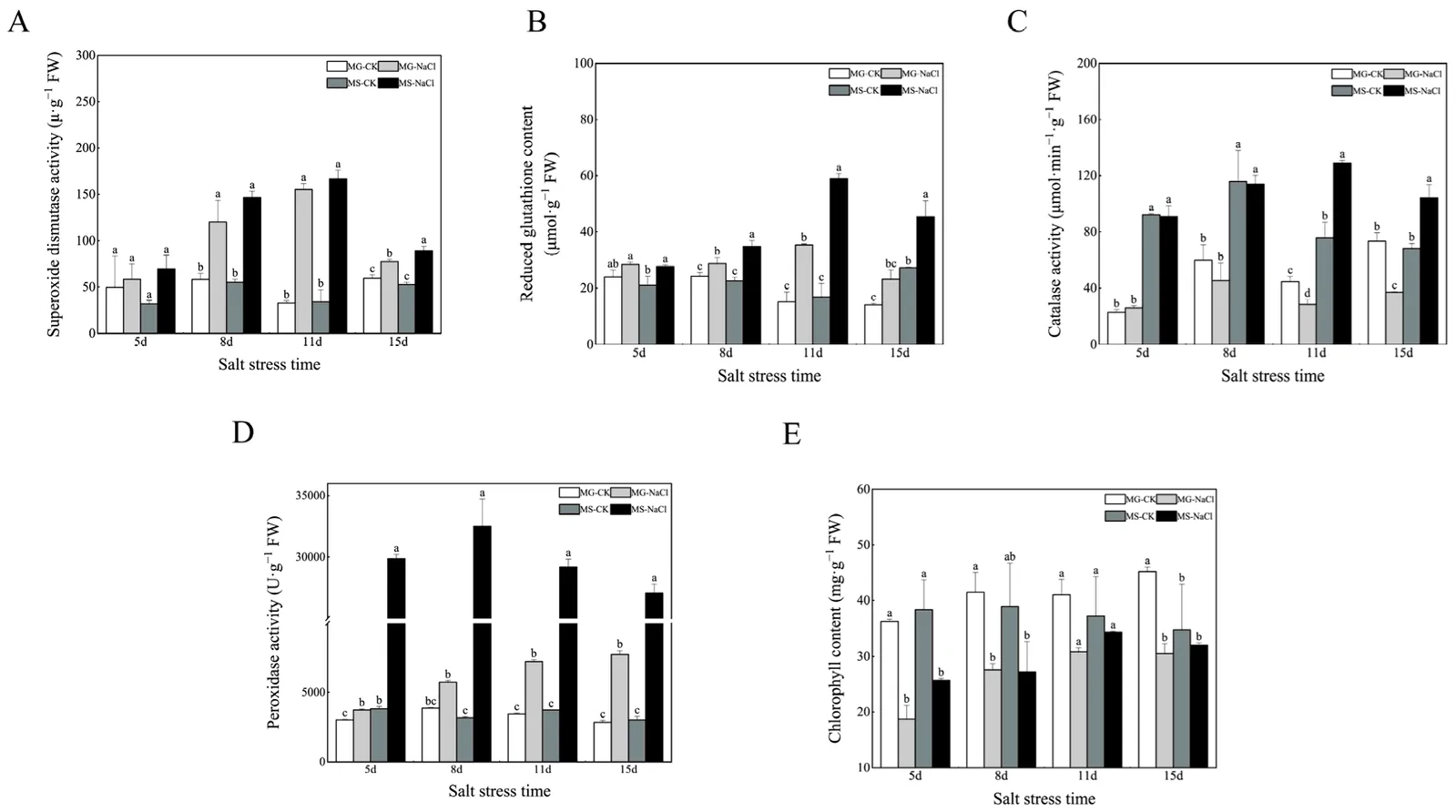
Changes in superoxide dismutase (SOD) activity (**A**), reduced glutathione (GSH) content (**B**), catalase (CAT) activity (**C**), peroxidase (POD) activity (**D**), and chlorophyll content (**E**) of two oat varieties (MS and MG) subjected to salt stress for 5, 8, 11, and 15 d. Values are mean ± SD. Different lowercase letters indicate significant differences among the variety × salt treatment combinations within each sampling time point (*p* < 0.05).

The three-way ANOVA indicated that oat variety, salt treatment, and time had varying effects on antioxidant enzyme activities, with significant interactions among some factors (Table 2). Variety exerted highly significant effects on GSH content and POD activity (*p* < 0.001), whereas it had no significant effects on SOD and CAT activities. The variety × salt treatment interaction exerted a significant effect on SOD activity (*p* < 0.05), and highly significant effects on GSH content, CAT activity and POD activity (*p* < 0.001). Time exerted highly significant effects on SOD and CAT activities (*p* < 0.001), and significant effects on GSH content and POD activity (*p* < 0.05). The salt treatment × time interaction produced highly significant effects on SOD, GSH and CAT (*p* < 0.001), and a significant effect on POD activity (*p* < 0.05). The variety × time interaction exerted highly significant effects on GSH and POD (*p* < 0.001), and a significant effect on CAT (*p* < 0.05), but had no significant effect on SOD. The variety × salt treatment × time interaction had a significant effect on GSH (*p* < 0.05), and highly significant effects on CAT and POD (*p* < 0.001). Under salt stress, SOD activity, CAT activity, and GSH content in both MG and MS showed an overall trend of initial increase followed by decrease (Figure 3A–D). MS generally exhibited higher SOD, CAT, and POD activities, as well as GSH content, than MG, with the greatest difference observed for POD activity under salt stress. Except at 5 d, SOD activity differed significantly between the salt-treated and corresponding CK groups in both varieties (*p* < 0.05). Under salt stress, SOD activity reached its maximum at 11 d relative to the CK, with increases of 78.90% in MG and 79.42% in MS. GSH content also peaked at 11 d under salt stress compared with CK, increasing by 57.16% in MG and 71.31% in MS. For CAT activity under severe salt stress, MS achieved the maximum increase of 77.94% at 11 d compared with CK, while MG reached its peak increment of 75.21% at 5 d. POD activity in MS peaked at 8 d with an 81.15% increase, while MG showed its greatest increase of 63.26% at 15 d under salt stress.

#### 2.3.2. Changes in Chlorophyll Content

The three-way ANOVA revealed that salt treatment exerted a highly significant effect on chlorophyll content (*p* < 0.001), while time and the variety × salt treatment interaction showed significant effects (*p* < 0.05). The salt treatment × time interaction and other remaining factors and interactions had no significant effects (Table 2). Under salt stress, chlorophyll content was lower in salt-stressed MS and MG plants than in their corresponding CK groups at all sampling time points (Figure 3E). Relative to their corresponding CK groups, chlorophyll content decreased by 24.81%—48.30% in MG and by 7.82%—33.18% in MS. However, there were no significant differences between MS and MG across all sampling time points under salt stress.

#### 2.3.3. Changes in Osmotic Adjustment Substances

**Figure 4:**
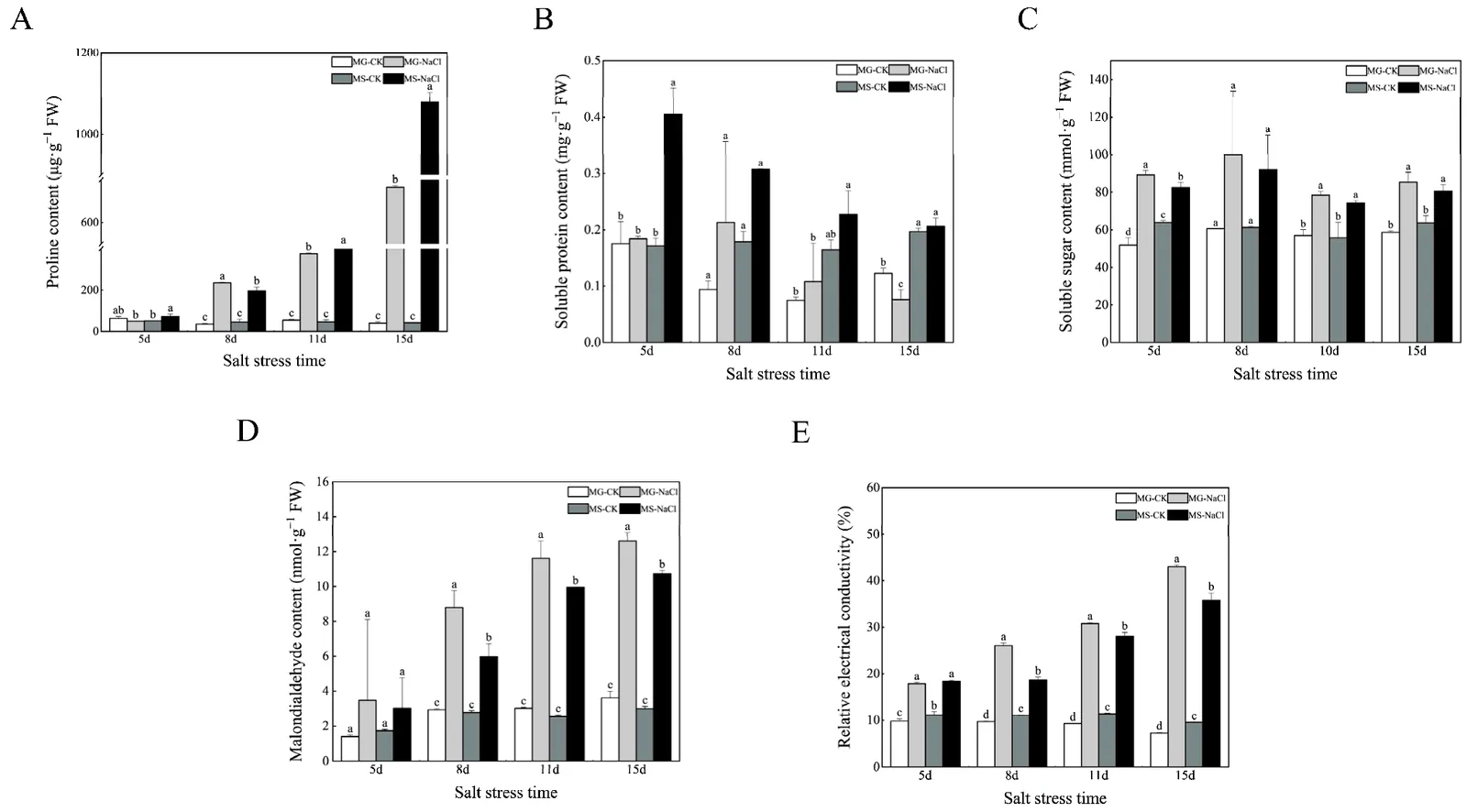
Changes in proline (PRO) content (**A**), soluble protein (SP) content (**B**), soluble sugar (SS) content (**C**), malondialdehyde (MDA) content (**D**), and relative electrolyte conductivity (REC) (**E**) in two oat varieties (MS and MG) subjected to salt stress for 5, 8, 11, and 15 d. Values are mean ± SD. Different lowercase letters indicate significant differences among the variety × salt treatment combinations within each sampling time point (*p* < 0.05).

The three-way ANOVA revealed that variety, salt treatment, time, and all their interactions exerted highly significant effects on proline (PRO) content (*p* < 0.001) (Table 3). For soluble protein (SP) content, variety and salt treatment exerted highly significant effects (*p* < 0.001), while time showed a significant effect (*p* < 0.05). The variety × salt treatment and salt treatment × time interactions significantly influenced SP content (*p* < 0.05), whereas the variety × time and three-way interaction had no significant effects. Soluble sugar (SS) content was only highly significantly affected by salt treatment (*p* < 0.001); variety, time and all interaction terms showed no significant influences on SS content. Under salt stress, PRO content increased rapidly with increasing duration of salt stress in both varieties. Significant differences between the salt-stressed and CK groups were observed in MG at all sampling time points except day 5. At 15 d, PRO content reached its maximum in both varieties, increasing by 102.91% in MG and 154.68% in MS relative to 11 d. SP content exhibited different response trends between the two varieties, decreasing progressively in MS but increasing initially before declining in MG under salt stress. Compared with the corresponding CK groups, SP content increased by 28.34% in MS at 5 d and decreased by 4.78% in MG at 15 d, with significant differences observed only at these time points (*p* < 0.05). Overall, SP content was generally higher in MS than in MG under salt stress. SS content in both varieties showed an initial increase, followed by a decrease and a subsequent increase during salt stress. Compared with the corresponding CK groups, SS content was significantly higher at all sampling time points (*p* < 0.05). The greatest increase was observed in MG at 5 d, reaching 72.31%, and MG accumulated significantly more SS than MS at this time point (Figure 4A–C).

#### 2.3.4. Changes in Cell Membrane Permeability

The three-way ANOVA revealed that variety, salt treatment, time, and their interactions all had highly significant effects on relative electrolyte conductivity (REC) (*p* < 0.001) (Table 3). For MDA content, salt treatment, time, and the salt treatment × time interaction had highly significant effects (*p* < 0.001), whereas variety had a significant effect (*p* < 0.05). Under salt stress, both MDA content and REC increased progressively in the two oat varieties, whereas only minor changes were observed under normal growth conditions. Across all sampling time points, salt-treated MG plants generally exhibited higher MDA content and REC than salt-treated MS plants, with significant differences observed at all time points except 5 d (*p* < 0.05). At 15 d of salt stress, MDA content reached its maximum, with increases of 68.60% in MG and 78.96% in MS relative to the corresponding CK levels. REC also peaked at 15 d, showing an increase of 16.48% in MG and 26.55% in MS compared with the corresponding CK values (Figure 4D,E).

### 2.4. Transcriptomic Analysis of Salt-Stressed Oats

#### 2.4.1. Raw Data Quality Assessment

**Figure 5:**
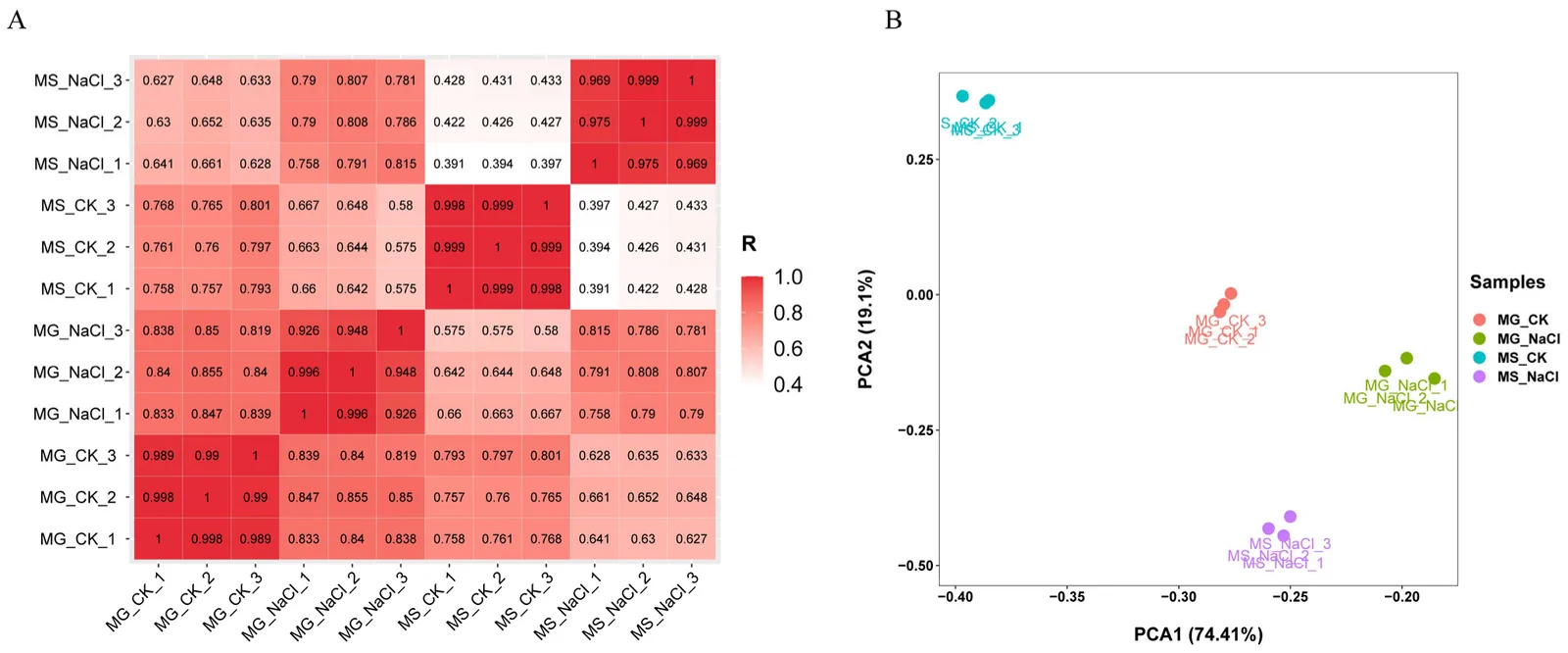
(**A**) Pearson correlation heatmap among samples. (**B**) Principal component analysis (PCA) of samples.

To investigate the transcriptomic responses to salt stress, leaf samples of MS and MG were collected under control conditions (normal growth for 15 days) and salt stress (300 mM NaCl for 15 days). A total of 12 samples were sequenced, generating 4.89 × 10^8^ raw reads (Table S9). Q20 and Q30 reached 99.62% and 97.18%, respectively, indicating high sequencing quality. After data filtering and quality control, 4.68 × 10^8^ clean reads were retained, with an average of 3.90 × 10^7^ clean reads per library. Correlation coefficients among replicates within each treatment group were all above 0.8 and consistently higher than those between different groups (Figure 5A,B), demonstrating good biological reproducibility and confirming the reliability of the transcriptome sequencing data.

#### 2.4.2. Screening for Differentially Expressed Genes (DEGs) in Oats

**Figure 6:**
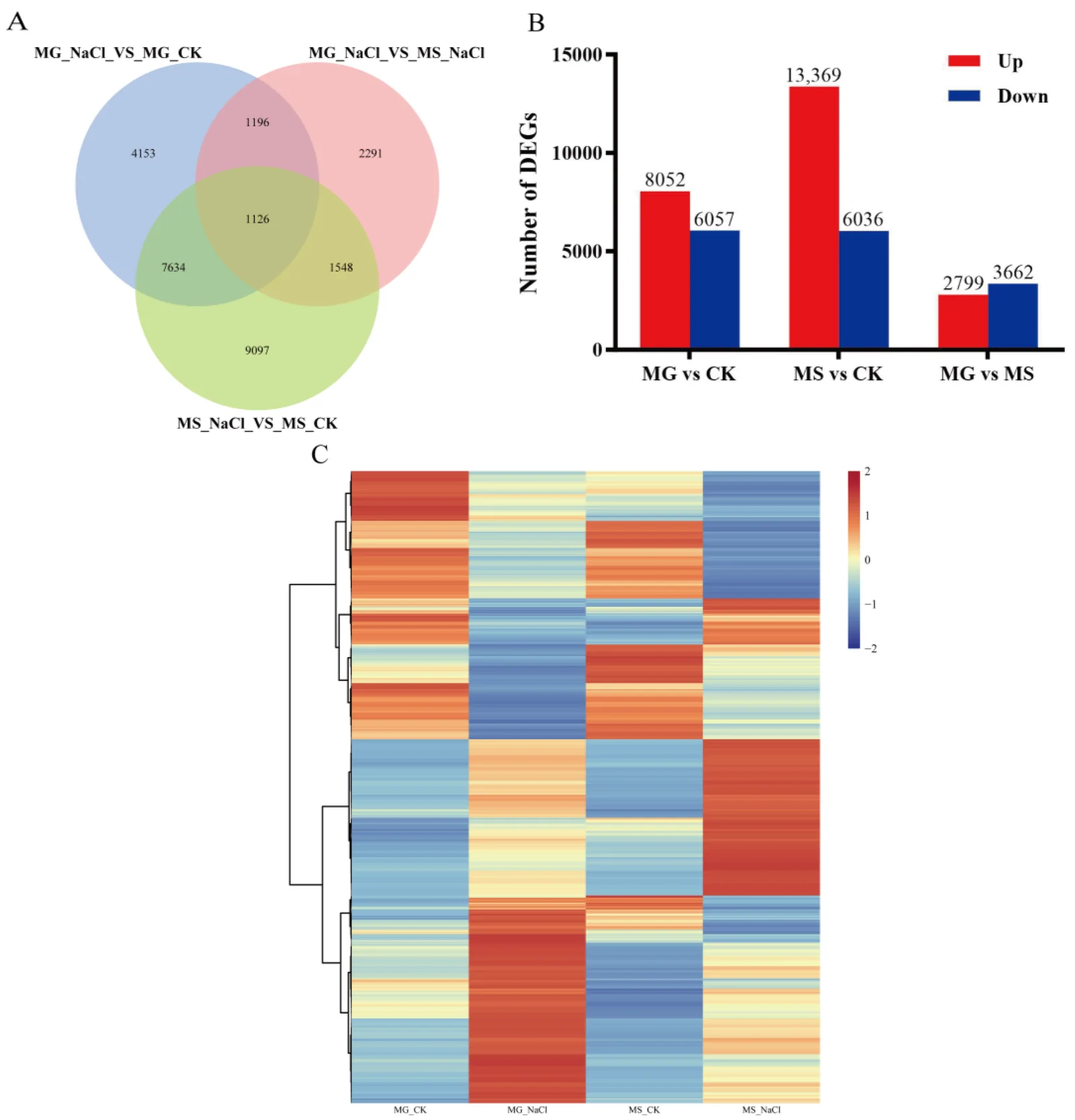
(**A**) Venn diagram of overlapping DEGs. (**B**) Numbers of DEGs. (**C**) Hierarchical clustering analysis of DEGs.

Transcriptome data from CK and salt-stressed samples of both MG and MS after 15 days of stress were analyzed using DESeq2. The outcomes highlighted 14,109 DEGs in the MG vs. CK comparison (8052 upregulated and 6057 downregulated), 19,405 DEGs in the MS vs. CK comparison (13,369 upregulated and 6036 downregulated), and 6161 DEGs in the MG vs. MS comparison (2799 upregulated and 3362 downregulated) (Figure 6B). A Venn diagram illustrated the distribution of DEGs across the three comparisons (Figure 6A). A total of 1126 genes were shared by all three comparisons, implying that these core genes might perform vital functions in the salt stress response of oats. Hierarchical clustering analysis revealed clear differences between MG and MS (Figure 6C).

#### 2.4.3. Gene Ontology (GO) Enrichment Analysis

To investigate the functional categories of DEGs under salt stress, GO enrichment analysis was performed on the DEGs from the three comparisons. In the MG vs. CK, MS vs. CK, and MG vs. MS comparisons, 430, 477, and 168 GO terms were significantly enriched, respectively (*p* < 0.05), with a total of 421 unique GO terms identified across all three comparisons. In the biological process category, the enriched terms across the three comparisons were primarily associated with protein phosphorylation, transmembrane transport, translation, carbohydrate metabolic process, and regulation of DNA-templated transcription. In the cellular component category, the enriched terms included membrane, ribosome, and nucleus. In the molecular function category, the enriched terms included protein binding, ATP binding, protein kinase activity, and structural constituent of ribosome. These results indicate that under salt stress, both varieties respond through common pathways, including signal transduction, transmembrane transport, protein synthesis, and energy metabolism (Figure S1).

#### 2.4.4. Kyoto Encyclopedia of Genes and Genomes (KEGG) Pathway Enrichment Analysis

**Figure 7:**
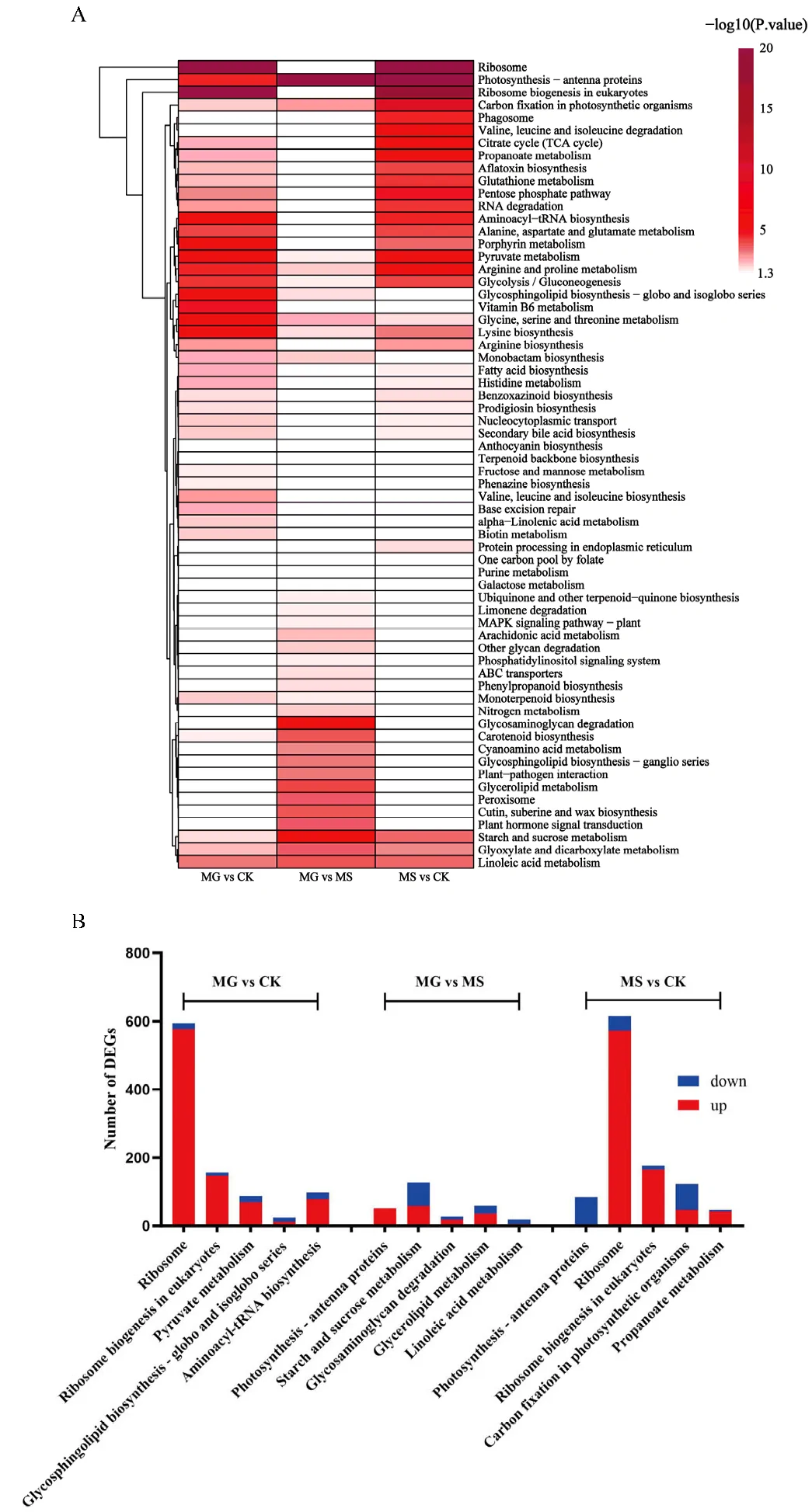
(**A**) Heatmap depicting significant KEGG pathways (*p* < 0.05) across comparisons. (**B**) Top five KEGG pathways enriched among DEGs.

A total of 64 KEGG pathways were significantly enriched in at least one comparison (*p* < 0.05) (Figure 7A), with the MG vs. CK group exhibiting the highest number of enriched pathways. Across the three comparisons, 12 metabolic pathways were commonly enriched in all three comparisons. Seventeen pathways were uniquely enriched in the MG vs. MS comparison, potentially reflecting differential responses between the two varieties. Enriched pathways were ranked according to their *p*-values. Among the top five KEGG pathways, the ribosome pathway and ribosome biogenesis in eukaryotes were significantly enriched in both the MG vs. CK and MS vs. CK comparisons (Figure 7B), but did not differ significantly between the two varieties. In contrast, the photosynthesis-antenna protein pathway was enriched in both the MS vs. CK and MG vs. MS comparisons, indicating a differential response between the salt-tolerant and salt-sensitive varieties.

#### 2.4.5. Starch and Sucrose Metabolism Pathways

**Figure 8:**
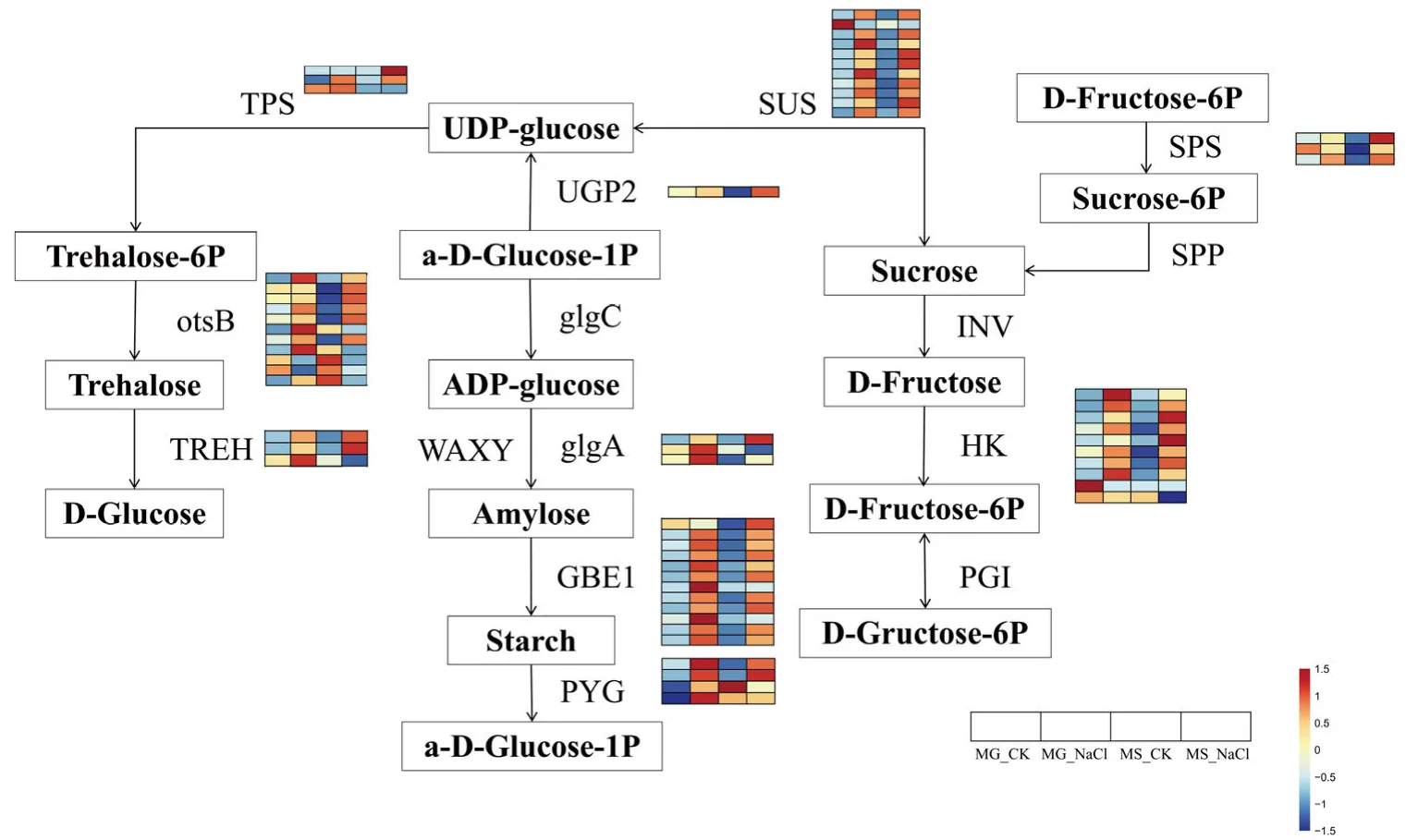
Starch and sucrose metabolism pathway in response to salt stress.

Starch and sucrose metabolism provides the core carbon sources for plant growth, development, and stress adaptation, and this pathway was significantly enriched in all three comparisons. Key genes within the pathway were selected for expression pattern analysis. Under salt stress, most genes involved in sucrose metabolism (*SPS*, *SUS*), trehalose synthesis (*TPS*, *otsB*, *TREH*), and starch metabolism (*glgA*, *GBE1*, *PYG*) were significantly upregulated in both MS and MG. Overall, the magnitude of upregulation was higher in MS than in MG (Figure 8).

#### 2.4.6. Plant Hormone Signaling Pathways

**Figure 9:**
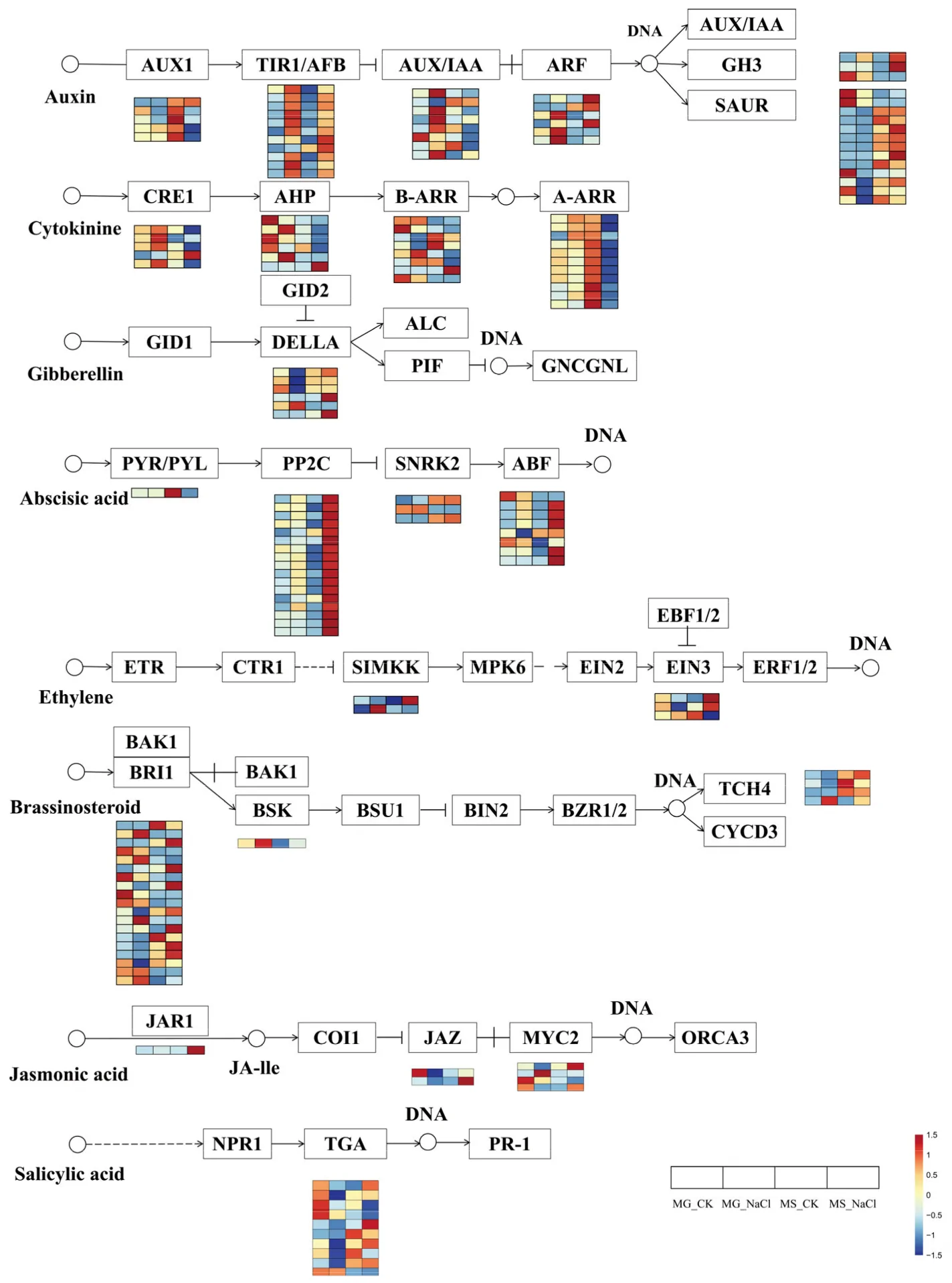
Transcriptional patterns of genes involved in plant hormone signal transduction under salt stress conditions.

Plant hormone signaling pathways were enriched in both MG and MS during salt stress conditions. In the MS variety, several hormone signaling pathways associated with stress resistance underwent significant changes. In the abscisic acid (ABA) pathway, downstream components, including *SnRK2* and the transcription factor *ABF*, were upregulated, whereas the upstream receptor *PYR/PYL* did not show a consistent upregulation trend, with some genes exhibiting downregulation. In the jasmonic acid (JA) pathway, *JAZ*-related genes were upregulated, while *MYC2*-related genes exhibited a mixed expression pattern, with both upregulation and downregulation. In the salicylic acid (SA) pathway, *TGA*-related genes showed expression changes, with some genes being upregulated. Additionally, in MS, certain hormone pathways associated with growth and development—such as the auxin, cytokinin, and gibberellin pathways—did not exhibit a consistent overall downregulation trend. Instead, differential expression was observed among different components. In the auxin pathway, expression changes in *AUX1*, *TIR1*/*AFB*, *AUX*/*IAA*, and *ARF*-related genes were inconsistent, with some genes showing both upregulation and downregulation. However, most of their downstream response genes, such as *GH3* and *SAUR*, were significantly upregulated. In the cytokinin pathway, expression changes in *AHP* and *B-ARR*-related genes were inconsistent, whereas *A*-*ARR* showed a significant downregulation trend. In the gibberellin pathway, *DELLA* genes exhibited an upregulated expression pattern, except for those that remained unchanged. However, their associated regulatory components did not show a consistent trend. In contrast, the hormone response in MG under salt stress displayed a different pattern, with most genes showing either downregulation or no change (Figure 9).

#### 2.4.7. qRT-PCR Analysis

Ten DEGs with high expression levels and large fold changes were chosen for real-time quantitative PCR (qRT-PCR) analysis. Although the fold changes observed by qRT-PCR differed slightly from those detected by RNA-seq, the overall expression trends were largely consistent, indicating that the transcriptomic data are reliable (Figure S2).

### 2.5. Weighted Gene Co-Expression Network Analysis

#### 2.5.1. WGCNA Module Analysis

To identify genes potentially involved in salt tolerance, WGCNA was performed. In this analysis, genes were grouped into seventeen modules (Figure S3A). A topological overlap matrix (TOM) heatmap revealed that genes clustered into distinct co-expression modules (Figure S3B). The module-sample correlation analysis showed that the yellow module was significantly positively correlated with the salt-treated MS (*p* < 0.01) and showed no significant correlation with other groups, suggesting that this module represents a differential response between MG and MS (Figure S3C). Furthermore, the yellow module exhibited highly significant positive correlations with physiological indices related to antioxidant defense and osmotic regulation, including PRO, GSH, POD, SOD and CAT (Figure S3D). Collectively, these findings indicate that the yellow module is closely associated with salt stress adaptation and antioxidant defense processes. Therefore, the yellow module was selected for subsequent analysis.

#### 2.5.2. Hub Gene Screening

**Figure 10:**
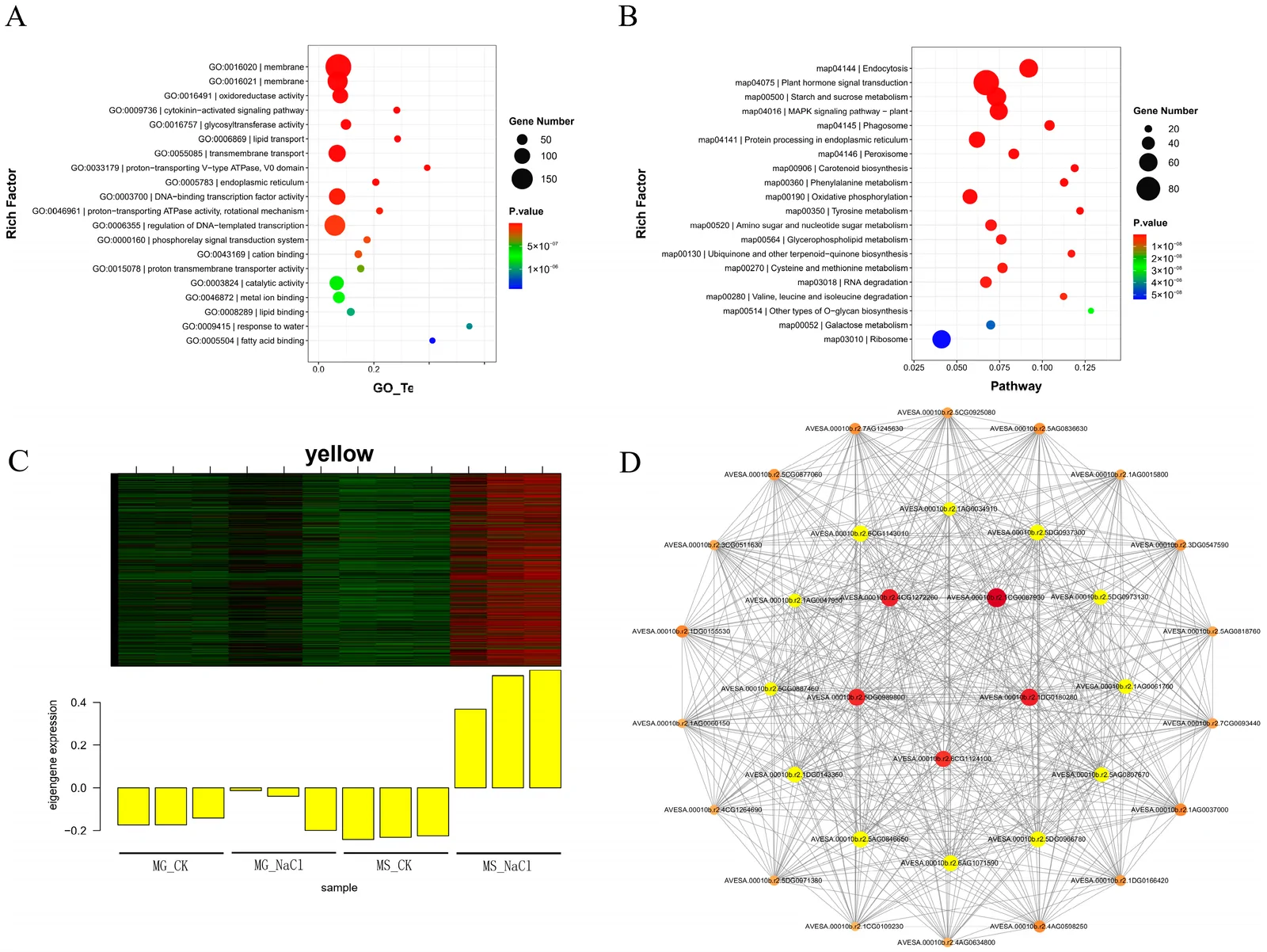
(**A**) GO enrichment analysis of the yellow module. (**B**) KEGG pathway enrichment analysis of the yellow module. (**C**) Gene expression pattern of the yellow module. (**D**) Hub genes in the yellow module.

Genes in the yellow module were subjected to GO enrichment analysis, which revealed significant enrichment in molecular functions including proton-transporting ATPase activity, transmembrane transport, lipid transport, and metal ion binding, as well as biological processes such as regulation of DNA-templated transcription and phosphorelay signal transduction system (Figure 10A). KEGG pathway enrichment analysis revealed that the genes in this module were primarily associated with various metabolic and signaling pathways, including plant hormone signal transduction, starch and sucrose metabolism, the plant MAPK signaling pathway, and other related pathways (Figure 10B). Analysis of the gene expression pattern in the yellow module revealed that genes within this module were significantly upregulated in MS compared to MG (Figure 10C). Using Cytoscape software for hub gene screening, five hub genes were identified as *AVESA.00010b.r2.1CG0087930* (*MGL*), *AVESA.00010b.r2.19DG0180280* (*MGL*), *AVESA.00010b.r2.4CG1272260* (*BCH1*), *AVESA.00010b.r2.5DG0989800* (*GPAT7*), and *AVESA.00010b.r2.6CG1124100* (*TPR10*) (Figure 10D). Their roles in salt tolerance in oats require further verification.

## 3. Discussion

### 3.1. Screening for Salt Tolerance in Oat Varieties During the Germination Stage

The ability of plants to survive and complete their life cycle under saline–alkali stress conditions depends on their salt tolerance, which varies among species and developmental stages [23]. Seed germination is highly sensitive to various environmental factors, including salinity, drought, and extreme temperatures [24,25]. Morphological traits, such as GR and root length, are considered reliable indicators for identifying salt-tolerant crops [26,27]. In the current study, 28 oat varieties were evaluated using five relative indicators: GR, GE, GI, root length, and shoot length. Principal component analysis and hierarchical clustering of these traits grouped the varieties into four categories, identifying MS as salt-tolerant and MG as salt-sensitive.

### 3.2. Responses of Oat Growth and Physiological Parameters Under Salt Stress

The effects of oat variety, salt treatment, and time differed among the measured growth and physiological traits, with some parameters showing significant main effects or interaction effects. Similar approaches have been increasingly applied in plant stress studies, where multiple factors are considered to better understand variations in physiological responses [28]. In the present study, salt treatment significantly affected all measured growth and physiological parameters, indicating that severe NaCl stress was the dominant factor influencing early seedling performance. Under these conditions, salt stress caused changes in multiple physiological processes, including reduced growth, altered chlorophyll content, impaired membrane stability, osmotic adjustment, and antioxidant responses.

Plant phenotypes serve as direct responses to environmental stress, with morphological changes reflecting the severity of stress damage [29]. This study found that salt stress significantly impacted both MS and MG phenotypes. As the duration of stress increased, both oat varieties were affected; however, MS remained relatively erect throughout, while MG suffered more severe damage. This observation agrees with the findings of Derbali, who reported that salt-tolerant varieties can maintain normal morphology by regulating physiological metabolism, while sensitive varieties are more prone to damage, with symptoms worsening over time under prolonged stress [30]. Plant height, fresh weight, and dry weight are key indicators of plant growth [31]. As the duration of salt stress increased, plant height in both varieties showed an upward trend, although the growth rate was significantly lower than that of the CK group. The reduction in plant height was greater in MG than in MS, suggesting that the two varieties exhibited different degrees of growth inhibition under the tested salt conditions. Salt stress affects the plant photosynthetic system, and changes in chlorophyll content serve as a primary indicator of damage to the photosynthetic system [32]. Husain et al. conducted a study on six durum wheat (*Triticum turgidum* L. subsp. *durum*) genotypes with varying salt-tolerance levels [33]. The results showed that, compared to salt-tolerant wheat, the decline in chlorophyll content in the leaves of salt-sensitive wheat was more pronounced. Similarly, research by Akca and Samsunlu indicated that chlorophyll levels in walnut (*Juglans regia* L.) leaves decreased significantly after salt stress, with the reduction being more pronounced in salt-sensitive varieties [34]. In this study, chlorophyll content was measured in two oat varieties under salt stress. Both varieties showed a decreasing trend compared to the control group, but the reduction was greater in the salt-sensitive variety, indicating that salt stress adversely affects chlorophyll status in oats, with a relatively weaker effect on the salt-tolerant variety. The smaller reduction in chlorophyll content observed in MS suggests that the salt-tolerant variety maintained relatively greater chlorophyll stability under severe salt stress. Regarding membrane damage indicators, both REC and MDA are key stress response markers in plants under stress, and their levels are commonly used to indicate the degree of stress experienced by plants [35]. When exposed to salt stress, ionic toxicity and oxidative damage disrupt cell membrane structure, leading to increased membrane permeability and elevated specific electrical conductivity, while simultaneously intensifying lipid peroxidation in cell membranes and causing MDA accumulation [36,37]. This study found that MDA levels rose significantly, with sensitive varieties showing a more rapid increase and higher overall levels.

Under salinity conditions, plants accumulate osmotic regulators such as PRO, SS and SP to lower cellular osmotic pressure, enhance water uptake capacity, maintain turgor pressure, and alleviate osmotic stress damage [38]. Proline levels in the leaves of both oat varieties increased in response to salt stress, with a more pronounced rise observed in MS leaves. These results suggest that osmotic adjustment, particularly proline accumulation, contributes to the response of oat seedlings to salt stress. Salt stress induces the production of large amounts of ROS in plant cells; excessive accumulation of ROS can damage cell structure and function, leading to cell injury or even death [39]. Plants mitigate oxidative damage by activating antioxidant systems to scavenge excess ROS [40]. As a key defense mechanism for plant adaptation to salt stress, the strength of antioxidant system activity directly reflects a plant’s salt tolerance [41]. Sreenivasalu et al. found that under salt stress, the activities of SOD and APX increased in seedlings of salt-tolerant lines, whereas both were inhibited in salt-sensitive lines [42]. Exposure to salt stress enhanced the activities of SOD, CAT, and POD and increased GSH concentrations in both MS and MG, while the magnitude of changes in antioxidant-related parameters differed between the two varieties during early seedling responses to severe salt stress, with MS exhibiting more pronounced antioxidant responses.

### 3.3. Transcriptomic Response of Oats

Salt stress induces systemic changes in plants, in which transcriptional regulation plays a central role in stress-responsive processes. GO enrichment analysis of DEGs in this study revealed that DEGs in both varieties were primarily concentrated in processes such as protein phosphorylation, transmembrane transport, translation regulation, and carbohydrate metabolism. KEGG enrichment analysis further indicated significant enrichment in categories such as Arginine and Proline Metabolism, Photosynthesis-Antenna Proteins and Starch and Sucrose Metabolism in both varieties. It has been demonstrated in previous studies that numerous pathways are linked to plant salt tolerance [43,44]. Comparative transcriptomic analysis of salt-tolerant and salt-sensitive rice (*Oryza sativa* L.) showed that sucrose and starch metabolism was markedly responsive to salt stress, and two genes involved in sucrose biosynthesis were significantly upregulated [45]. Plants quickly modulate carbohydrate and energy metabolic pathways to supply energy for coping with salinity stress [46]. Studies in oats have also highlighted that starch and sucrose metabolism pathways are key to responding to salinity stress [47]. In this study, the significant enrichment of starch and sucrose metabolism pathways across the three comparisons is consistent with these findings. Genes involved in the synthesis of sugars such as sucrose and trehalose were significantly upregulated in both MG and MS.

Plant hormone signaling pathways are important components of plant responses to abiotic stress. Studies have demonstrated that multiple phytohormone pathways participate in rice salt stress responses. For example, *OsNCED3* and *OsNCED5* enhance salt tolerance by regulating plant hormone pathways [48,49]. In MS, core components of the ABA, BR, JA, and SA signaling pathways (*BRI1*, *BZR1/2*, *COI1*, *JAZ*, some *MYC2*, and *TGA*) were significantly upregulated, indicating altered transcriptional regulation of hormone signaling-related genes under salt stress. Concurrently, in MS, key genes in growth-related hormone pathways, including auxin, cytokinin, and gibberellin (such as *AUX1*, *TIR1*/*AFB*, *ARF*, *AHP*, *B-ARR*, and *DELLA*), exhibited a mixed pattern of upregulation and downregulation, reflecting complex regulation of hormone-related processes under salt stress. In MG, hormone signaling-related genes also showed distinct expression patterns under salt stress, with differences observed among different hormone pathways.

### 3.4. Analysis of Hub Genes via WGCNA

To date, various plant species have been analyzed using WGCNA [22,50,51]. The yellow module, which exhibited an extremely significant positive correlation with the salt-tolerant variety MS, was selected for further investigation. Given the relatively limited number of transcriptomic samples, WGCNA was considered an exploratory approach to identify candidate co-expression modules associated with salt stress responses rather than to establish direct causal relationships. The KEGG enrichment analysis highlighted that genes within the yellow module are strongly linked to plant hormone signaling pathways. Combined with previous analyses, these findings suggest that hormone-related transcriptional regulation may contribute to the differences in salt stress responses between the two varieties. Further analysis of the gene interaction network within the yellow module identified five candidate hub genes: *AVESA.00010b.r2.1CG0087930* (*MGL*), *AVESA.00010b.r2.19DG0180280* (*MGL*), *AVESA.00010b.r2.4CG1272260* (*BCH1*), *AVESA.00010b.r2.5DG0989800* (*GPAT7*), and *AVESA.00010b.r2.6CG1124100* (*TPR10*). In plants, methionine γ-lyase (MGL) is one of the key enzymes involved in methionine catabolism. Methionine contributes significantly to plant metabolism because it serves as the precursor for S-adenosylmethionine, which in turn functions as the precursor for the biosynthesis of ethylene, polyamines, biotin, and other essential metabolites [52]. In *Arabidopsis thaliana*, *AtMGL* expression was induced by drought and salt stresses, suggesting that MGL contributes to osmotic stress tolerance by promoting isoleucine biosynthesis as an osmoprotectant [53,54,55]. BCH, a key enzyme in the β-branch of carotenoid biosynthesis, functions upstream of ABA biosynthesis [56,57]. Carotenoids can effectively scavenge ROS, participate in photosynthesis, and provide substrates for ABA biosynthesis [58]. In *Lycium chinense*, the *BCH* gene enhanced carotenoid accumulation and improved salt tolerance in transgenic tobacco (*Nicotiana tabacum*) plants [59]. A β-carotene hydroxylase gene (*DSM2*) in rice plays a key role in stress resistance by modulating the xanthophyll cycle and endogenous ABA levels, particularly in response to drought and oxidative stress [60]. GPATs are key enzymes involved in glycerolipid biosynthesis. Previous studies have demonstrated that *GPAT* genes are closely associated with stress tolerance and oil accumulation [61,62]. In *Suaeda salsa*, heterologous overexpression of *SsGPAT* increased resistance to salt stress in *Arabidopsis*, stabilizing membrane structure, alleviating oxidative damage, and reducing ion toxicity under salt stress [63]. *TaGPAT6* confers enhanced resistance to salinity in wheat (*Triticum aestivum*) by promoting the accumulation of cutin and suberin [64]. Likewise, soybean (*Glycine max* (L.) Merr.) hairy roots with overexpressed *GmGPAT1* showed promoted root elongation, greater biomass accumulation and elevated photosynthetic capacity when exposed to 120 mM NaCl [65]. Tetratricopeptide repeat (TPR) proteins mediate protein–protein interactions in diverse biological processes, and the interaction of *SlTPR1* with *NR* and *LeETR1* suggests its role in ethylene- and auxin-regulated pathways [66,67]. Beyond hormone signaling, several TPR proteins have been reported to participate in stress regulation. For example, overexpression of *MdTPR16* reduced salt sensitivity in apple (*Malus domestica*) calli, apple seedlings, and rooted apple plantlets [68]. These previous studies provide functional references for interpreting the potential roles of the identified hub genes. However, considering that these candidates were identified based on co-expression relationships, their direct contributions to salt tolerance in oats remain to be experimentally validated through functional studies.

## 4. Materials and Methods

### 4.1. Material Preparations

Twenty-eight oat varieties commonly used in production and originating from different regions were selected as test materials. The seeds of all oat cultivars used in this study were provided by the Grassland Science Laboratory of Northeast Agricultural University. Detailed information on the cultivar names and seed sources is provided in Table S1. Seeds were surface-sterilized with 75% ethanol followed by 10% sodium hypochlorite solution, and rinsed three times with sterile distilled water. The sterilized seeds were placed in Petri dishes (230 mm × 190 mm) lined with three layers of moistened filter paper for germination. For each oat variety, two treatments (control and salt stress) were established, with four biological replicates for each variety × treatment combination. Each replicate corresponded to an independent Petri dish containing 30 uniformly sized oat seeds. To perform the salinity stress treatment, 10 mL of 250 mM NaCl solution was added to each dish, and the control samples were given the same amount of distilled water. To maintain consistent salinity levels and minimize evaporation, the dishes were sealed with parafilm, and no additional NaCl solution was added during the experiment. All Petri dishes were incubated at 25 °C and 60% relative humidity under full-spectrum white T8 LED plant-growth lamps (Zhongshan Senliang Lighting Co., Ltd., Zhongshan, China) at a light intensity of 6400 lx, with a 12 h light/12 h dark photoperiod. A seed was considered germinated when the radicle had emerged and reached at least 2 mm in length. Germination counts were performed at the same time daily up to seven d post-sowing. Three germination-related indices, GR, GE and GI, were calculated according to the daily germination records, with detailed formulas provided in Section 4.2. At seven days post-germination, ten seedlings with uniform growth were selected from each Petri dish (each biological replicate), and root and shoot lengths were measured with a ruler.

### 4.2. Preliminary Screening of Materials

The following indicators were calculated based on the measured data:

(1) $$\mathrm{Germination}\;\mathrm{rate}\;(\mathrm{GR})=(\frac{\mathrm{Number}\;\mathrm{of}\;\mathrm{germinated}\;\mathrm{seeds}\;\mathrm{on}\;\mathrm{day}\;7}{\mathrm{Total}\;\mathrm{number}\;\mathrm{of}\;\mathrm{tested}\;\mathrm{seeds}})\;\times \;100\%\;$$

(2) $$\mathrm{Germination}\;\mathrm{energy}\;(\mathrm{GE})=(\frac{\mathrm{Number}\;\mathrm{of}\;\mathrm{germinated}\;\mathrm{seeds}\;\mathrm{at}\;\mathrm{the}\;\mathrm{peak}\;\mathrm{germination}\;\mathrm{period}}{\mathrm{Total}\;\mathrm{number}\;\mathrm{of}\;\mathrm{tested}\;\mathrm{seeds}})\times 100\%\;$$

(3) $$\mathrm{Germination}\;\mathrm{index}\;(\mathrm{GI})=\sum \frac{G_{t}}{D_{t}}$$

*G_t_* represents the number of seeds that germinated on day *D_t_*.

(4) $$\mathrm{Relative}\;\mathrm{value}\;\mathrm{of}\;\mathrm{each}\;\mathrm{trait}:\frac{\mathrm{Trait}\;\mathrm{value}\;\mathrm{under}\;a\;\mathrm{given}\;\mathrm{salt}\;\mathrm{concentration}}{\mathrm{Trait}\;\mathrm{value}\;\mathrm{of}\;\mathrm{control}}\times \;100\%$$

(5) $$U(X_{j})=(X_{j}-X_{min})/(X_{max}-X_{min})$$

where *U*(*X_j_*) is the membership-function value of the *j*-th comprehensive index; *X_j_* is the value of the *j*-th comprehensive index, *X_max_* and *X_min_* are the maximum and minimum values, respectively.

(6) $$W_{j}=P_{j}/\sum_{j=1}^{n}P_{j}$$

where *W_j_* is the weight of the *j*-th comprehensive index; *P_j_* is the contribution rate of the *j*-th comprehensive index across all varieties.

(7) $$D=\sum_{j=1}^{n}\left[U(X_{j})\times W_{j}\right]$$

where *D* is the comprehensive evaluation value; *U*(*X_j_*) is the membership-function value of the *j*-th comprehensive index; *W_j_* denotes the weight of the *j*-th comprehensive index.

Comprehensive scores for the 28 varieties were calculated using PCA based on the relative values of the measured traits [69]. Hierarchical cluster analysis was then performed using squared Euclidean distance.

### 4.3. Experimental Treatments

Based on the germination screening of 28 oat varieties, MS was identified as salt-tolerant and MG as salt-sensitive. These two varieties were selected for subsequent experiments. Seeds were sown in rectangular plastic seedling pots (10 cm × 10 cm) filled with vermiculite, with five seedlings per pot and a total of 200 seedlings. The seedlings were cultivated in a growth chamber under controlled conditions (25 °C day/20 °C night, 12 h light/12 h dark photoperiod, 70% relative humidity, and illumination provided by full-spectrum white T8 LED plant-growth lamps at a light intensity of 6400 lx). After three weeks of growth, the seedlings were subjected to salt stress treatment. The control group received 20 mL of standard Hoagland’s nutrient solution every two days, whereas the salt-stressed group received an equal volume of Hoagland’s nutrient solution supplemented with 300 mM NaCl every two days. For each oat variety and treatment, three biological replicates were established, with each biological replicate consisting of three seedlings. Shoot tissues were collected at 5, 8, 11, and 15 d after treatment, immediately frozen in liquid nitrogen, and stored at −80 °C until further analysis.

### 4.4. Assessment of Growth and Physiological Responses to Salt Stress

The measured growth parameters included plant height, FW, and DW. Plant height was measured from the root collar to the plant apex using a ruler, and the fresh weight of the aboveground portion was recorded. Following fresh weight determination, the plants were dried at 105 °C for 30 min to deactivate enzymes and then maintained at 85 °C until a constant weight was achieved, after which the dry weight was measured. Chlorophyll content was determined using the 95% ethanol extraction method. Fresh leaf tissue (0.1 g) was extracted with 1 mL of 95% ethanol until completely decolorized, and the absorbance was measured at 663 and 645 nm for chlorophyll content determination. REC was measured using a conductivity meter (DDS-307-A, Ray Magnetic Instrument Co., Ltd., Shanghai, China). Fresh oat leaves were rinsed three to five times with deionized water and blotted dry. The leaves were cut along the veins and placed in 10 mL centrifuge tubes containing 8 mL of ultrapure water. After shaking for 30 min, the samples were left for 12 h, and the initial conductivity (L_1_) was measured. The samples were then heated in a water bath at 100 °C for 20 min and cooled to room temperature before the final conductivity (L_2_) was measured. The conductivity of ultrapure water was recorded as L_0_. REC was calculated as REC (%) = [(L_1_ − L_0_)/(L_2_ − L_0_)] × 100. MDA content, as an indicator of lipid peroxidation, was determined using the thiobarbituric acid colorimetric method [70]. Physiological parameters, including POD, SOD, and CAT activities, as well as PRO, GSH, SS and SP contents, were quantified using commercial assay kits (Suzhou Keming Biological Co., Ltd., Suzhou, China). The specific testing procedures followed the manufacturer’s instructions.

### 4.5. Transcriptomic Analysis

Transcriptome sequencing was performed using leaf tissues from the salt-sensitive oat variety MG and the salt-tolerant oat variety MS after 15 d of control conditions without NaCl addition (MG-CK and MS-CK) or NaCl-induced salt stress (MG-NaCl and MS-NaCl). Each oat variety × treatment combination included three biological replicates. Total RNA was extracted using TRIzol reagent (Cat. 15596018, Thermo Fisher, Waltham, MA, USA). RNA concentration, purity, and integrity were assessed using a Qubit 3.0 fluorometer (Cat. Q33216, Thermo Fisher, Waltham, MA, USA) and an Agilent 5300 Fragment Analyzer (Cat. M5311AA, Agilent, Santa Clara, CA, USA). Only RNA samples with an RNA Integrity Number (RIN) > 7.0 were used for library construction. Poly(A)+ mRNA was enriched from 2 μg of total RNA using mRNA Capture Beads 2.0 (Cat. 12629ES, Yeasen, Shanghai, China), fragmented, and reverse-transcribed into cDNA. Following second-strand cDNA synthesis, end repair, A-tailing, adapter ligation, PCR amplification, and size selection (approximately 400 bp), sequencing libraries were constructed and sequenced on an Illumina NovaSeq™ X Plus platform using a paired-end 2 × 150 bp strategy at LC-Bio Technology Co., Ltd. (Hangzhou, China). Raw reads were trimmed to remove adapters, poly-A/G tails, and low-quality sequences using Cutadapt (v1.9). The quality of the resulting clean reads was subsequently assessed using FastQC (v0.11.9). Clean reads were aligned to the oat reference genome using HISAT2 (v2.2.1). Transcript assembly was performed using StringTie (v2.1.6), followed by transcript merging using gffcompare (v0.9.8). Transcript abundance was estimated using StringTie and Ballgown, and expression levels were reported as fragments per kilobase of transcript per million mapped reads (FPKM). Differential expression analysis among the experimental groups was performed in R using the DESeq2 package. Genes with an absolute fold change ≥ 2 and a false discovery rate (FDR) < 0.05 were considered DEGs. GO and KEGG pathway enrichment analyses were subsequently performed for the identified DEGs. Pathways with a hypergeometric *p*-value < 0.05 were considered significantly enriched.

### 4.6. qRT-PCR

Based on the RNA-seq results, ten DEGs showing high expression levels and substantial fold changes were selected for qRT-PCR analysis. The same RNA samples used for RNA-seq were used for qRT-PCR analysis. qRT-PCR was performed in a 10 μL reaction volume. The primer sequences are listed in Table S6, and the detailed reaction mixtures and thermocycling conditions are provided in Tables S7 and S8. Amplification efficiencies of all primer pairs were determined using standard curves generated from serially diluted cDNA. No-template control (NTC) was included in each qRT-PCR run to detect potential reagent contamination. The oat *AsUBC* gene, selected based on previous studies, was used as the reference gene for normalization [71]. Relative expression levels were calculated using the 2^−ΔΔCt^ method. Each oat variety × treatment combination included three biological replicates. Amplification specificity was assessed by melting curve analysis.

### 4.7. WGCNA

WGCNA was performed using the OmicStudio platform (https://www.omicstudio.cn/tools, accessed on 14 February 2026) to construct the gene co-expression network. The analysis was based on the transcriptomic data described in Section 4.5, comprising 12 RNA-seq samples collected at 15 days after treatment. These samples included three biological replicates for each variety × salt treatment combination, including MG-CK and MS-CK under control conditions and MG-NaCl and MS-NaCl under salt stress conditions. Prior to network construction, genes with an FPKM < 2 across all samples were filtered out. Pearson correlation coefficients between genes were calculated using gene expression values of all individual biological replicates, and the correlation matrix was transformed into an adjacency matrix using a weighted network approach. The soft-thresholding power was selected as β = 12 according to the scale-free topology criterion, with the scale-free topology fit index (R^2^) threshold set at 0.85. The adjacency matrix was subsequently converted into a topological overlap matrix (TOM) to evaluate network connectivity. Gene modules were identified using a dynamic tree-cutting algorithm with a minimum module size of 30 genes, and highly similar modules were merged using a merge cut height of 0.25. Module eigengenes were calculated to evaluate the relationships between modules, samples, experimental conditions, and physiological traits. Pearson correlation analysis was performed to assess module–trait relationships, and the significance of correlations was determined based on correlation coefficients and *p*-values. Gene module visualization was performed using Cytoscape v3.9.1.

### 4.8. Data Analysis

The effects of oat variety, salt treatment, time, and their interactions on physiological traits were evaluated using a three-way analysis of variance (ANOVA) model. At each sampling time, one-way ANOVA followed by Duncan’s multiple range test was performed to compare differences among the four variety × salt treatment combinations (MG-CK, MG-NaCl, MS-CK, and MS-NaCl). Data were tested for normality and homogeneity of variance before ANOVA. Each variety × salt treatment combination consisted of three biological replicates. Statistical analyses were conducted using SPSS v. 19.0. Data visualization and figure generation were performed using Origin 2026 and R software (version 4.5.2).

## 5. Conclusions

This study screened 28 oat varieties at the germination stage under severe NaCl treatment and identified MS and MG. Using these two varieties, we systematically analyzed early-seedling physiological responses and transcriptomic alterations under severe NaCl stress treatment (300 mM NaCl). Salt stress markedly affected growth, chlorophyll content, osmotic adjustment, antioxidant enzyme activities, and gene expression, with MS showing better overall performance than MG under salt stress. WGCNA identified five hub genes that are potentially associated with salt stress responses in oats, which may provide preliminary references for future functional validation and breeding-related research.

## Figures and Tables

**Table 1:** Three-way ANOVA of the effects of oat variety, salt treatment, and time on plant height, fresh weight, and dry weight.

Measurement Indicators	F Value and Its Significance						
	Variety	Salt Treatment	Time	Variety × Salt Treatment	Variety × Time	Salt Treatment × Time	Variety × Salt Treatment × Time
Plant height	23.753 ***	187.664 ***	49.573 ***	5.193 *	0.984	19.339 ***	1.162
Fresh weight	488.603 ***	766.138 ***	107.153 ***	9.365 **	43.419 ***	124.628 ***	18.53 ***
Dry weight	217.866 ***	209.082 ***	46.923 ***	7.158 *	10.395 ***	7.722 **	2.059

Note: *, **, and *** indicate significance at *p* < 0.05, *p* < 0.01, and *p* < 0.001, respectively.

**Table 2:** Three-way ANOVA of the effects of oat variety, salt treatment, and time on SOD activity, CAT activity, GSH content, POD activity, and chlorophyll content.

Measurement Indicators	F Value and Its Significance						
	Variety	Salt Treatment	Time	Variety × Salt Treatment	Variety × Time	Salt Treatment × Time	Variety × Salt Treatment × Time
SOD	1.009	216.148 ***	24.83 ***	6.288 *	0.55	31.747 ***	0.299
GSH	64.324 ***	235.738 ***	7.245 *	28.394 ***	22.110 ***	34.405 ***	4.439 *
CAT	0.914	387.688 ***	13.775 ***	42.386 ***	3.938 *	8.571 ***	11.56 ***
POD	2352.912 ***	3531.905 ***	6.115 *	2294.175 ***	12.117 ***	3.725 *	14.767 ***
Chlorophyll	0.077	56.6 ***	3.936 *	5.112 *	1.817	1.798	0.542

Note: * and *** indicate significance at *p* < 0.05 and *p* < 0.001, respectively.

**Table 3:** Three-way ANOVA of the effects of oat variety, salt treatment, and time on PRO, SP, SS, MDA, and REC.

Measurement Indicators	F Value and Its Significance						
	Variety	Salt Treatment	Time	Variety × Salt Treatment	Variety × Time	Salt Treatment × Time	Variety × Salt Treatment × Time
PRO	135.041 ***	9427.425 ***	2468.736 ***	143.933 ***	113.604 ***	2570.229 ***	114.451 ***
SP	32.149 ***	14.739 ***	5.584 *	5.042 *	0.052	3.483 *	1.768
SS	0.049	38.371 ***	1.425	1.41	0.123	0.671	0.15
MDA	5.287 *	180.833 ***	28.104 ***	3.086	0.537	13.826 ***	0.234
REC	58.674 ***	11,806.992 ***	689.182 ***	342.768 ***	33.312 ***	1013.439 ***	38.314 ***

Note: * and *** indicate significance at *p* < 0.05 and *p* < 0.001, respectively.

## Data Availability

The RNA-seq datasets generated in this study have been deposited in the NCBI Sequence Read Archive under accession number PRJNA1464350. The seeds of the two tested oat varieties (MS and MG) are available from the corresponding author upon reasonable request.

## Supplementary Materials

The following supporting information can be downloaded at https://www.mdpi.com/article/10.3390/plants15172712/s1. Table S1: Oat varieties and sources tested; Table S2: Germination indices, root length and shoot length of 28 varieties under control and treatment conditions; Table S3: Relative values of germination indices of 28 oat varieties; Table S4: Component score coefficients, eigenvalues, and variance contribution rates from principal component analysis; Table S5: Coefficients, variance contribution rates, and *D*-values of the two comprehensive indicators derived from principal component analysis; Table S6: Primers used for qRT-PCR analysis; Table S7: qRT-PCR reaction system; Table S8: qRT-PCR thermal cycling conditions; Table S9: Summary of RNA-seq data; Figure S1: GO enrichment analysis of DEGs in the (A) MG vs. CK, (B) MG vs. MS, and (C) MS vs. CK comparisons; Figure S2: qRT-PCR validation of DEGs; Figure S3: (A) Module hierarchical clustering dendrogram. (B) Module correlation analysis. (C) Module–sample correlation analysis. (D) Module–physiological trait correlation analysis.
