# Supplementary material for: Integrative Physiological and Transcriptomic Analyses of Oat (Avena sativa L.) Seedlings Under Severe Salt Stress
Source: Plants (Basel). 2026 Sep 3;15(17):2712. doi: 10.3390/plants15172712 (PMC13567349; doi:10.3390/plants15172712)
Supplement: Supplementary file 1 [file plants-15-02712-s001.zip › Figure S1.pdf]

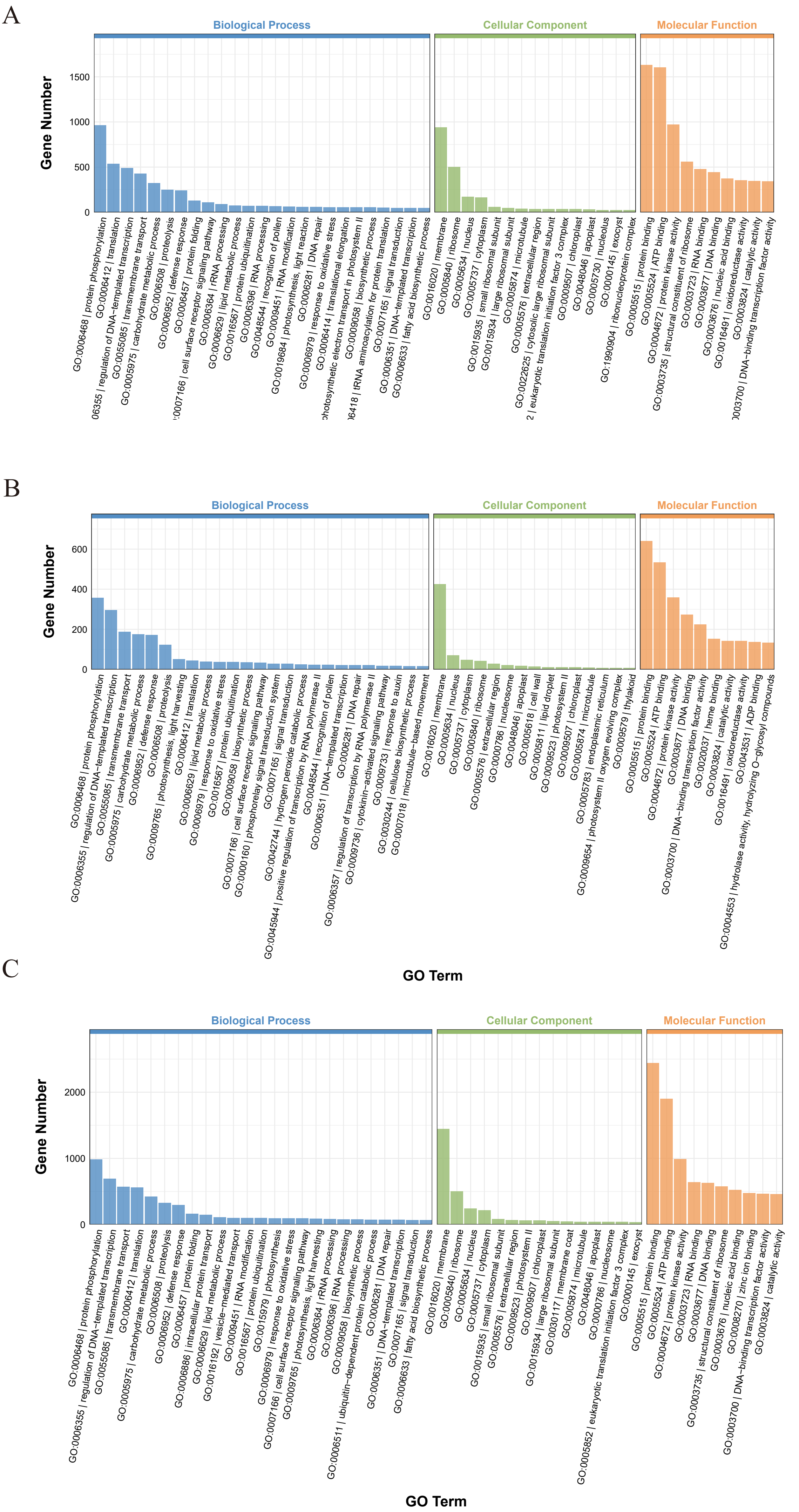

Figure S1. GO enrichment analysis of DEGs in the (A) MG vs CK, (B) MG vs MS, and (C) MS vs CK comparisons.
