# Supplementary material for: Integrative Physiological and Transcriptomic Analyses of Oat (Avena sativa L.) Seedlings Under Severe Salt Stress
Source: Plants (Basel). 2026 Sep 3;15(17):2712. doi: 10.3390/plants15172712 (PMC13567349; doi:10.3390/plants15172712)
Supplement: Supplementary file 1 [file plants-15-02712-s001.zip › Figure S3.pdf]

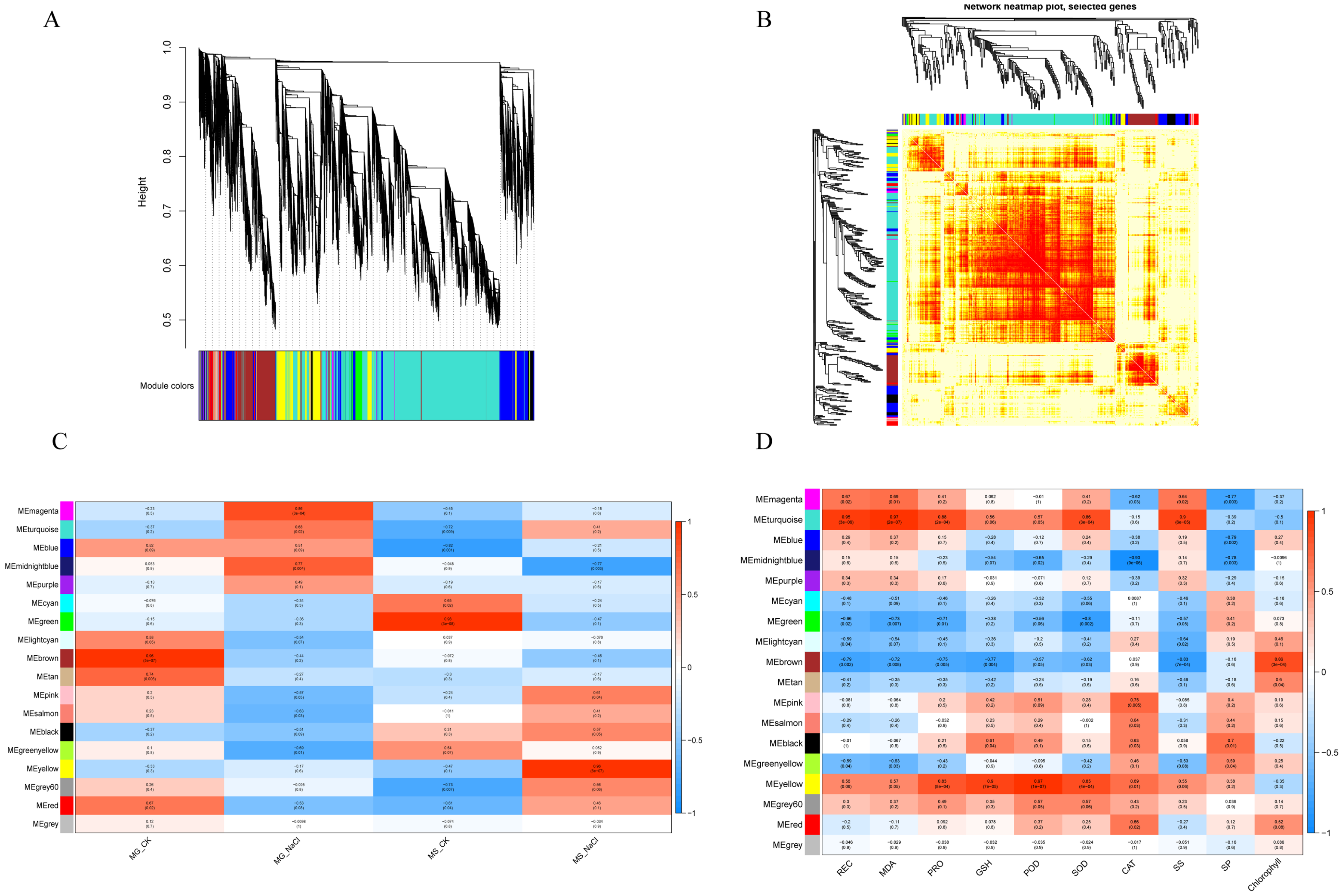

Figure S3. (A) Module hierarchical clustering dendrogram. (B) Module correlation analysis. (C) Module–sample correlation analysis. (D) Module–physiological trait correlation analysis.
