# Supplementary material for: Integrative Physiological and Transcriptomic Analyses of Oat (Avena sativa L.) Seedlings Under Severe Salt Stress
Source: Plants (Basel). 2026 Sep 3;15(17):2712. doi: 10.3390/plants15172712 (PMC13567349; doi:10.3390/plants15172712)
Supplement: Supplementary file 1 [file plants-15-02712-s001.zip › Table S1.pdf]

Table S1. Oat varieties and sources tested.

Breed	Variety acquisition channel
Surui	Sanfeng Landscape Co., Ltd.
Baylor	Sanfeng Landscape Co., Ltd.
Satsuma	Sanfeng Landscape Co., Ltd.
Suter	Sanfeng Landscape Co., Ltd.
Madison	Institute of Grassland Science, Heilongjiang Academy of Agricultural Sciences
Flag	Institute of Grassland Science, Heilongjiang Academy of Agricultural Sciences
Haymaker	Institute of Grassland Science, Heilongjiang Academy of Agricultural Sciences
Baylor II	Institute of Grassland Science, Heilongjiang Academy of Agricultural Sciences
Pony	Institute of Grassland Science, Heilongjiang Academy of Agricultural Sciences
Haywire	Institute of Grassland Science, Heilongjiang Academy of Agricultural Sciences
Mustang	Institute of Grassland Science, Heilongjiang Academy of Agricultural Sciences
Qingyin No.1	Qinghai Academy of Animal Husbandry and Veterinary Sciences
Morgan	Beijing Best Grass Industry Co., Ltd.
Yumu No.1	Beijing Best Grass Industry Co., Ltd.
Modern	Beijing Best Grass Industry Co., Ltd.
Dictator	Beijing Zhengdao Seed Industry Co., Ltd.
Monida	Beijing Zhengdao Seed Industry Co., Ltd.
Mengshi No.1	Beijing Mammoth Seed Company
Sanxing	Beijing Mammoth Seed Company
Magnum	Xi’an Bailv Landscape Design Co., Ltd.
Sweet Oats No.70	Clover (Beijing) Ecological Technology Co., Ltd.
Souris	Clover (Beijing) Ecological Technology Co., Ltd.
Muda	Clover (Beijing) Ecological Technology Co., Ltd.
Sweet Oats No.60	Beijing Baiqingyuan Animal Husbandry Technology Development Co., Ltd.
Kona	Beijing Baiqingyuan Animal Husbandry Technology Development Co., Ltd.
Sweet Oats No.75	Beijing Baiqingyuan Animal Husbandry Technology Development Co., Ltd.
Fuyan No.2	Beijing Baiqingyuan Animal Husbandry Technology Development Co., Ltd.
Fuyan No.60	Beijing Baiqingyuan Animal Husbandry Technology Development Co., Ltd.
