# Supplementary material for: Integrative Physiological and Transcriptomic Analyses of Oat (Avena sativa L.) Seedlings Under Severe Salt Stress
Source: Plants (Basel). 2026 Sep 3;15(17):2712. doi: 10.3390/plants15172712 (PMC13567349; doi:10.3390/plants15172712)
Supplement: Supplementary file 1 [file plants-15-02712-s001.zip › Table S2.pdf]

Table S2. Germination indices, root length and shoot length of 28 varieties under control and treatment conditions.

Variety	Germination rate (%)		Germination energy (%)		Germination index		Root length (cm)		Shoot length (cm)	
	CK	T	CK	T	CK	T	CK	T	CK	T
Surui	87.5±1.6	55±2.89	80.84±3.44	34.17±6.49	59.41±2.13	15.17±1.3	8.6±0.38	0.61±0.05	8.16±0.36	0.41±0.03
Souris	72.5±3.16	39.17±5.99	59.08±2.45	28.33±2.15	39.1±3.49	18.45±2.2	9.39±0.24	0.8±0.04	9.31±0.15	1.3±0.13
Baylor	95±1.67	24.17±5.67	70±7.58	18.33±7.27	50.92±2	4.95±1.24	10.2±0.26	0.26±0.04	10.42±0.33	0.17±0.04
Kona	89.17±2.85	62.5±0.83	84.17±3.15	42.58±4.41	61.11±2.87	30.11±1.87	10.24±0.28	0.78±0.04	8.74±0.2	1.34±0.12
Madison	95±2.15	80±4.08	88.34±4.19	55.83±3.7	62.48±3.37	41.05±1.81	11.07±0.49	1.06±0.04	8.86±0.38	0.89±0.06
Modern	86.67±2.36	69.17±2.85	77.84±0.79	49.17±3.44	55.99±1	37.65±2.22	10.55±0.4	0.8±0.08	9.89±0.24	0.8±0.09
Satsuma	99.17±0.83	70.83±6.44	93.33±1.36	55±9.12	69.84±1.16	31.87±3.42	9.7±0.27	0.79±0.05	9.45±0.26	1.37±0.21
Sweet oats No.70	80±1.36	53.33±1.36	60±7.07	44±1.91	39.98±2.03	20.04±1.77	8.54±0.39	0.91±0.07	8.29±0.22	1.15±0.15
Magnum	88.33±3.97	72.5±2.85	77.5±3.94	45±2.15	58.04±3.57	35.13±1.73	9.56±0.24	0.66±0.04	8.82±0.3	0.58±0.06
Flag	89.17±2.1	77.5±2.5	79.92±4.28	50.83±3.63	57.43±1.96	34.96±2.29	9.18±0.27	1.02±0.04	10.1±0.25	1.26±0.09
Muda	83.34±4.51	80.84±2.5	80.88±3.22	65.84±3.15	61.04±2.85	47.76±1.95	9±0.25	0.48±0.04	9.21±0.32	0.42±0.06
Qingyin No.1	80.83±1.59	70±4.51	78.34±0.96	51.75±8.35	55.5±1.08	34.37±4.01	10.34±0.52	1.15±0.07	11.24±0.3	1.67±0.08
Morgan	90±2.72	8.33±2.15	70±5.93	7±1.78	44.11±1.61	1.33±0.52	10.63±0.22	0.42±0.05	9.38±0.31	0.3±0.03
Fuyan No.60	70.84±7.86	58.33±1.67	53.5±6.42	39.17±0.83	39.13±4.51	27.96±1.25	9.33±0.25	0.55±0.04	9.45±0.32	0.64±0.08
Yumu No. 1	95.83±1.6	48.33±5.69	88.33±2.89	35±4.97	61.56±2.35	12.96±2.18	10.69±0.35	0.64±0.05	11.37±0.4	0.43±0.04
Dictator	80.83±7.12	56.67±9.13	75.83±7.62	42.5±6.44	54.46±5.05	28.95±4.36	10.19±0.26	0.84±0.05	10.15±0.26	0.69±0.14
Haywire	87.5±5.51	64.17±6.44	86.67±6.09	50.83±6.29	64±3.37	32.43±4.07	8.83±0.46	0.84±0.07	11.86±0.12	0.94±0.09
Mengshi No. 1	98.34±0.96	88.34±3.47	92.33±3.79	80±2.36	68.14±1.56	57.74±1.29	10.6±0.3	1.02±0.06	10.9±0.32	0.87±0.07
Baylor II	96.67±2.36	65±0.96	87.5±3.8	40.08±2.41	59.29±2.74	29.36±1.5	10.88±0.39	0.7±0.04	9.73±0.36	0.47±0.04
Sweet oats No.60	75±5.53	65.84±5.16	63.42±5.68	50.92±3.98	45.12±3.68	34.71±2.04	9.19±0.38	0.43±0.03	9.3±0.22	0.47±0.04
Pony	94.17±2.85	70.83±7.86	91±3.45	50.83±9.27	67.42±2.32	35.32±4.79	8.9±0.24	0.53±0.06	8.49±0.16	0.33±0.03
Monida	93.33±0	83.33±5.44	93±0.33	61±1.53	71.39±0.59	45.32±1.7	8.6±0.38	0.26±0.02	9.43±0.2	0.25±0.02
Suter	83.34±6.53	64.17±4.79	76.67±6.27	51.58±4.74	57.02±5.04	37.99±2.1	9.19±0.4	1.19±0.07	10.42±0.25	0.97±0.09
Haymaker	68.33±5.18	55±3.97	62.59±3.39	47.58±1.58	46.49±3.04	31.34±2.54	10.43±0.26	0.88±0.05	11.21±0.24	0.91±0.06
Mustang	76.67±7.33	65±2.15	75.83±7.53	54.67±2.57	57.18±5.9	38.69±1.64	9.83±0.28	0.92±0.05	10.53±0.3	0.43±0.02
Sweet oats No.75	75.25±2.84	65±3.37	60.25±4.15	30.75±1.44	42.27±2.74	23.37±0.47	9.7±0.38	0.55±0.04	8.69±0.16	0.52±0.1
Fuyan No.2	90±1.92	75.84±3.94	84.17±2.5	60±5.15	62.75±2.36	29.59±1.24	11.43±0.45	0.58±0.03	10.78±0.28	0.39±0.02
Sanxing	88.33±2.15	12.5±1.6	65±3.97	8.5±0.65	44.91±1.81	3.72±0.96	11.32±0.36	0.37±0.05	10.37±0.27	0.35±0.05

Note:Values are mean ± SE.
