# Supplementary material for: Integrative Physiological and Transcriptomic Analyses of Oat (Avena sativa L.) Seedlings Under Severe Salt Stress
Source: Plants (Basel). 2026 Sep 3;15(17):2712. doi: 10.3390/plants15172712 (PMC13567349; doi:10.3390/plants15172712)
Supplement: Supplementary file 1 [file plants-15-02712-s001.zip › Table S3.pdf]

Table S3. Relative values of germination indices of 28 oat varieties.

Variety	Germination rate (%)	Germination energy (%)	Germination index (%)	Root length (%)	Shoot length (%)
Surui	62.86	42.27	25.53	7.09	5.02
Souris	54.02	47.95	47.18	8.52	13.96
Baylor	25.44	26.07	9.73	2.55	1.63
Kona	70.09	50.59	49.28	7.62	15.33
Madison	84.21	63.21	65.7	9.58	10.05
Modern	79.81	63.17	67.25	7.58	8.09
Satsuma	71.43	58.93	45.64	8.14	14.5
Sweet oats No.70	66.67	73.33	50.12	10.66	13.87
Magnum	82.08	58.06	60.53	6.9	6.58
Flag	86.92	63.61	60.87	11.11	12.48
Muda	97	81.4	78.25	5.33	4.56
Qingyin No.1	86.6	66.06	61.92	11.12	14.86
Morgan	9.26	10	3.02	3.95	3.2
Fuyan No.60	82.35	73.21	71.45	5.89	6.77
Yumu No. 1	50.43	39.62	21.06	5.99	3.78
Dictator	70.1	56.04	53.15	8.24	6.8
Haywire	73.33	58.65	50.68	9.51	7.93
Mengshi No. 1	89.83	86.64	84.74	9.62	7.98
Baylor II	67.24	45.81	49.52	6.43	4.83
Sweet oats No.60	87.78	80.29	76.94	4.68	5.05
Pony	75.22	55.86	52.39	5.96	3.89
Monida	89.29	65.59	63.49	3.02	2.65
Suter	77	67.28	66.63	12.95	9.31
Haymaker	80.49	76.03	67.43	8.44	8.12
Mustang	84.78	72.09	67.66	9.36	4.08
Sweet oats No.75	86.38	51.04	55.29	5.67	5.98
Fuyan No.2	84.26	71.29	47.15	5.07	3.62
Sanxing	14.15	13.08	8.28	3.27	3.38
