# Supplementary material for: Integrative Physiological and Transcriptomic Analyses of Oat (Avena sativa L.) Seedlings Under Severe Salt Stress
Source: Plants (Basel). 2026 Sep 3;15(17):2712. doi: 10.3390/plants15172712 (PMC13567349; doi:10.3390/plants15172712)
Supplement: Supplementary file 1 [file plants-15-02712-s001.zip › Table S4.pdf]

Table S4. Component score coefficients, eigenvalues, and variance contribution rates from principal component analysis.

Indicators	Principal components				
	**PC1**	**PC2**	**PC3**	**PC4**	**PC5**
Relative root length	0.209	0.489	-1.462	0.202	-0.049
Relative shoot length	0.162	0.621	1.293	-0.058	0.061
Relative germination rate	0.273	-0.264	0.2	2.589	0.747
Relative germination energy	0.278	-0.232	0.028	-1.884	2.233
Relative germination index	0.279	-0.237	0.123	-0.773	-2.961
Eigenvalue	3.335	1.245	0.259	0.092	0.07
Contribution rate (%)	66.698	24.891	5.178	1.836	1.397
Cumulative contribution rate (%)	66.698	91.59	96.767	98.603	100
