# Supplementary material for: Integrative Physiological and Transcriptomic Analyses of Oat (Avena sativa L.) Seedlings Under Severe Salt Stress
Source: Plants (Basel). 2026 Sep 3;15(17):2712. doi: 10.3390/plants15172712 (PMC13567349; doi:10.3390/plants15172712)
Supplement: Supplementary file 1 [file plants-15-02712-s001.zip › Table S5 .pdf]

Table S5. Coefficients, variance contribution rates, and *D*-values of the two comprehensive indicators derived from principal component analysis.

Variety	X_1	X_2	U_1	U_2	*D*-value	Rank
Mengshi No. 1	1.29	-0.43	1	0.43	0.67	1
Qingyin No.1	1.03	1.42	0.93	0.95	0.62	2
Flag	0.89	1.1	0.9	0.86	0.6	3
Suter	0.92	0.97	0.9	0.83	0.6	4
Muda	0.74	-1.68	0.86	0.07	0.57	5
Madison	0.7	0.44	0.85	0.68	0.56	6
Sweet oats No.70	0.67	1.47	0.84	0.97	0.56	7
Haymaker	0.7	-0.19	0.85	0.5	0.56	8
Mustang	0.62	-0.64	0.82	0.37	0.55	9
Sweet oats No.60	0.56	-1.59	0.81	0.1	0.54	10
Fuyan No.60	0.48	-0.9	0.79	0.3	0.53	11
Modern	0.43	-0.19	0.78	0.5	0.52	12
Satsuma	0.28	1.27	0.74	0.91	0.49	13
Haywire	0.22	0.46	0.72	0.68	0.48	14
Kona	0.18	1.38	0.71	0.94	0.47	15
Magnum	0.19	-0.43	0.71	0.43	0.47	16
Dictator	0.03	0.1	0.67	0.58	0.45	17
Monida	-0.04	-1.95	0.65	0	0.44	18
Fuyan No.2	-0.03	-1.25	0.66	0.2	0.44	19
Souris	-0.07	1.58	0.65	1	0.43	20
Sweet oats No.75	-0.05	-0.65	0.65	0.37	0.43	21
Pony	-0.22	-0.81	0.61	0.32	0.4	22
Baylor II	-0.43	-0.33	0.55	0.46	0.37	23
Surui	-0.79	0.19	0.46	0.6	0.3	24
Yumu No. 1	-1.18	0.02	0.36	0.56	0.24	25
Baylor	-2.19	-0.34	0.09	0.45	0.06	26
Sanxing	-2.41	0.36	0.03	0.65	0.02	27
Morgan	-2.54	0.62	0	0.73	0	28
