# Supplementary material for: Integrative Physiological and Transcriptomic Analyses of Oat (Avena sativa L.) Seedlings Under Severe Salt Stress
Source: Plants (Basel). 2026 Sep 3;15(17):2712. doi: 10.3390/plants15172712 (PMC13567349; doi:10.3390/plants15172712)
Supplement: Supplementary file 1 [file plants-15-02712-s001.zip › Table S6.pdf]

Table S6. Primers used for qRT-PCR analysis.

Gene ID	Primer sequence（5′-3′）
AVESA.00010b.r2.4AG0613310	F:GCCGAGAACCAGAAGGTCAT<br>R:GGGCTGACATAGAACCCACC
AVESA.00010b.r2.2DG0395530	F:GTTACATTGACGCCTCCAACT<br>R:CACTAGCAGGCCTATCAAGC
AVESA.00010b.r2.1AG0057720	F:GGCTAAGGCTCCAGATGGTC<br>R:TGTACACGATGTCCGGGTTG
AVESA.00010b.r2.5DG1000030	F:CTCACTGTTGTGGAGGCCAT<br>R:ATGTGGAAACCGGACACTCC
AVESA.00010b.r2.5CG0887520	F:GTACAGCAGGACAACGATGC<br>R:GGTGCCCTTCTCCAAAGGAT
AVESA.00010b.r2.7AG1211440	F:ACCATGGCCACTCTATGTCG<br>R:GGCCAATACCAGCTTCCTGA
AVESA.00010b.r2.3DG0538230	F:CCCGTGGAGTGTTCACAGAA<br>R:GTTCCCGTATGCCTTGAGGT
AVESA.00010b.r2.6AG1061700	F:ACTACCGGAGGGCCATCTAC<br>R:CGTACTTCTCGCCGAGTGTT
AVESA.00010b.r2.1DG0150250	F:CAGGCTCAGGAAGGAGTGC<br>R:CTGGTTGTAGACGTAGGTGAGG
AVESA.00010b.r2.6CG1122490	F:GCAAGAGGGAGAAGACCAGG<br>R:AGTTCATGGCTGACCTTGGG
Oat Ubc	F:ACAGCGTTCCGAGAGTTGTT<br>R:AAGAGTCAGCCCGAATGCAG
