# Supplementary material for: Integrative Physiological and Transcriptomic Analyses of Oat (Avena sativa L.) Seedlings Under Severe Salt Stress
Source: Plants (Basel). 2026 Sep 3;15(17):2712. doi: 10.3390/plants15172712 (PMC13567349; doi:10.3390/plants15172712)
Supplement: Supplementary file 1 [file plants-15-02712-s001.zip › Table S7.pdf]

Table S7. qRT-PCR reaction system.

Components	Loading volume(μL)
2×Cham Q SYBR qPCR Master Mix	5
Forward primer(10 μmol·L-1)	0.4
Reverse primer(10 μmol·L-1)	0.4
cDNA	0.5
ddH2O	3.7
