# Supplementary material for: Integrative Physiological and Transcriptomic Analyses of Oat (Avena sativa L.) Seedlings Under Severe Salt Stress
Source: Plants (Basel). 2026 Sep 3;15(17):2712. doi: 10.3390/plants15172712 (PMC13567349; doi:10.3390/plants15172712)
Supplement: Supplementary file 1 [file plants-15-02712-s001.zip › Table S8.pdf]

Table S8. qRT-PCR thermal cycling conditions.

Temperature	Time	Cycles
95 ℃	3 min	
95 ℃	10 sec	
56 - 59 ℃	30 sec	40 cycles
72 ℃	30 sec	
Melting curve:		
56 - 59 ℃	15 sec	
95 ℃	60 sec	
