# Supplementary material for: Integrative Physiological and Transcriptomic Analyses of Oat (Avena sativa L.) Seedlings Under Severe Salt Stress
Source: Plants (Basel). 2026 Sep 3;15(17):2712. doi: 10.3390/plants15172712 (PMC13567349; doi:10.3390/plants15172712)
Supplement: Supplementary file 1 [file plants-15-02712-s001.zip › Table S9.pdf]

Table S9. Summary of RNA-seq data.

Sample	Raw Data	Valid Data	Valid Ratio（reads）	Q20%	Q30%	GC content%
MG_CK_1	45628264	44088776	96.63	99.63	97.26	51.50
MG_CK_2	32899228	31516654	95.80	99.63	97.36	51.50
MG_CK_3	36067748	34534014	95.75	99.59	97.25	52
MG_NaCl_1	40962216	39196050	95.69	99.63	97.11	51.50
MG_NaCl_2	42549472	41225700	96.89	99.61	97.35	51
MG_NaCl_3	47120570	46049210	97.73	99.55	96.46	49
MS_CK_1	41962562	38423486	91.57	99.65	97.58	55
MS_CK_2	41471768	38950606	93.92	99.64	97.48	55
MS_CK_3	42313328	39916768	94.34	99.64	97.52	55
MS_NaCl_1	32302690	30896258	95.65	99.57	96.91	50.50
MS_NaCl_2	38039908	36585024	96.18	99.63	96.77	52.50
MS_NaCl_3	48567272	47090236	96.96	99.64	97.09	52
Total	489885026	468472782	—	—	—	—
Average	40823752	39039399	95.59	99.62	97.18	52.21
